# Isolation, characterization, and pathogenicity of a *Vibrio parahaemolyticus* strain causing translucent post-larvae disease in *Penaeus vannamei* outside China

**DOI:** 10.1371/journal.pone.0331862

**Published:** 2025-09-15

**Authors:** Nguyen Dinh-Hung, Hung N. Mai, Maiya Matthews, Harris Wright, Arun K. Dhar

**Affiliations:** 1 Aquaculture Pathology Laboratory, School of Animal and Comparative Biomedical Sciences, The University of Arizona, Tucson, Arizona, United States of America; 2 Shrimp Improvement Systems, Islamorada, Florida, United States of America; National Cheng Kung University, TAIWAN

## Abstract

Translucent post-larvae disease (TPD) has emerged as a severe threat to shrimp aquaculture, causing substantial economic losses. The causative agent, *Vibrio parahaemolyticus*, has been primarily identified in China, but this study provides the first confirmed report of its presence in shrimp populations outside China. This research characterizes *V. parahaemolyticus* strain AG1 (*V*_*pTPD*_ AG1), isolated from diseased *Penaeus vannamei*, through biochemical, molecular, and pathogenic analyses. PCR screening of *V*_*pTPD*_ AG1 detected *vhvp-1* and *vhvp-2*, genes previously linked to TPD virulence, while *pirA/pirB* genes associated with acute hepatopancreatic necrosis disease (AHPND) were absent. Experimental immersion challenges demonstrated high virulence and dose-dependent pathogenicity, with an LC_50_ of 8.51 × 10^2^ CFU/mL at 96 hours in 15-day-old post-larvae (PL15) of *Penaeus vannamei* shrimp. Larger post-larvae (PL30) exhibited reduced susceptibility, suggesting a size-dependent resistance mechanism. Histopathological analysis confirmed the degeneration of the hepatopancreas, including tubular necrosis, epithelial cell sloughing, and bacterial invasion, consistent with previously reported TPD pathology. Additionally, hemocytic enteritis, a characteristic histopathological feature associated with infection with *V*_*pTPD*_ AG1 strain, was marked by mucosal epithelium loss, intense inflammation, and a thick hemocyte layer in the intestine. Antibiotic susceptibility testing of *V*_*pTPD*_ AG1 strain revealed resistance to β-lactams but sensitivity to multiple other antimicrobial classes. These findings highlight the expanding geographical distribution of *V*_*pTPD*_, its distinct pathological features compared to AHPND, and further highlight the urgent need to enhance surveillance and implement effective biosecurity measures to prevent its global dissemination.

## 1. Introduction

Shrimp aquaculture is a key sector of global seafood production, with *Penaeus vannamei* being the most widely farmed species due to its rapid growth, high adaptability, and economic value [1]. However, intensified farming practices have increased susceptibility to infectious diseases, with bacterial pathogens, particularly *Vibrio* species, posing a persistent and widespread threat to aquaculture sustainability [2,3]. Among these, *Vibrio parahaemolyticus* has been identified as the causative agent of several severe shrimp diseases, including acute hepatopancreatic necrosis disease (AHPND) and, more recently, translucent post-larvae disease (TPD). First reported in China [4], TPD has rapidly spread across shrimp-producing regions, raising serious biosecurity concerns regarding its potential for global dissemination and the significant risks it poses to shrimp aquaculture [5,6].

Translucent post-larvae disease is a highly lethal infectious disease affecting *P. vannamei* post-larvae, characterized by high mortality rates and distinct pathological manifestations, including a translucent body, pale hepatopancreas, an empty digestive tract, and severe histopathological damage [4–6]. The causative agent of TPD has been identified as a hypervirulent strain of *V. parahaemolyticus* (*V*_*pTPD*_), distinct from strains responsible for AHPND [7]. Unlike AHPND-associated *V. parahaemolyticus* (*V*_*pAHPND*_), which harbors a ~ 70 kb plasmid encoding the *Photorhabdus* insect-related (*pir*) *pirA/pirB* binary toxin [8], *V*_*pTPD*_ exhibits a unique pathogenicity mechanism driven by a novel set of virulence factors. Among these, *Vibrio* high virulent proteins (*vhvps*), particularly *vhpv-2*, carried on a 187,791 bp plasmid have been identified as the primary determinant of lethality [7]. Experimental challenge studies have demonstrated that *vhpv-2* is essential for disease manifestation, as its deletion [7] or the use of *V*_*pTPD*_ strains lacking this gene [5], significantly reduces pathogenicity in infected shrimp. Additional virulence factors, including *vhpv-1* and *vhpv-3*, have been identified on the same plasmid, suggesting a complex pathogenic mechanism [5]; however, their precise roles remains unknown. Despite initial reports of TPD being confined to China, emerging evidence indicates that *Vibrio* strains carrying TPD-associated virulence genes have been detected in other shrimp-producing regions [9]. The potential for horizontal gene transfer among *Vibrio* spp., combined with the global trade of live shrimp post-larvae, raises possibility of spreading this disease beyond its current geographical boundary and becoming a serious biosecurity concern. Additionally, environmental factors such as gut microbiota dysbiosis and pond water microbial imbalances may contribute to TPD outbreaks [10]. Given the rapid expansion of shrimp farming, the risk of TPD becoming a globally distributed disease necessitates urgent research to further characterize the pathogen, develop an optimized protocol for experimental bioassays in screening genetic lines of *P. vannamei*, validate diagnostic methods, and understand its transmission dynamics to prevent further spread.

This study presents the first confirmed isolation and and characterization of a *V. parahaemolyticus* strain associated with TPD from *P. vannamei* farmed outside China, marking a significant advancement in understanding the geographic expansion of this emerging pathogen. Through an integrated approach involving molecular detection, biochemical profiling, antibiotic susceptibility testing, experimental infection trials, and histopathological examinations, we delineate the characteristics and assess the pathogenicity of the *V*_*pTPD*_ strain now emerging beyond the geographic boundary of China. These findings provide an early warning of the risk of global dissemination and offer critical insights to support the development of effective surveillance, biosecurity, and disease management strategies aimed at safeguarding the sustainability of shrimp aquaculture worldwide.

## 2. Materials and methods

### 2.1. Sample collection, transport, and bacterial isolation

In October 2023, the Aquaculture Pathology Laboratory received shrimp samples for routine disease diagnosis from an anonymous hatchery in a Southeast Asian country outside China (exact location withheld at the farm’s request). The hatchery was experiencing high mortality among *P. vannamei* post-larvae (PL), with cumulative mortality exceeding 70% over a 5-day period. The outbreak began suddenly and progressed rapidly, prompting urgent diagnostic investigation. The clinical signs of diseased shrimp were consistent with previously reported cases of TPD, characterized by an abnormal hepatopancreas and pale, colorless digestive tracts, causing the body to become transparent. Representative diseased post-larvae were freshly collected at the hatchery and shipped in double-bagged, oxygenated source pond water placed within insulated, temperature-controlled polystyrene containers, in accordance with standard practice for live aquatic-animal transport. On receipt, bag integrity and PL condition were qualitatively documented. To minimize potential carryover from transport water, PL were rinsed in sterile seawater prior to necropsy. All diagnostic procedures were performed exclusively on tissues under sterile technique, and processing commenced immediately upon arrival. For bacterial isolation, pooled shrimp were homogenized in 1.0 mL of sterile saline solution, and the supernatant was serially diluted in sterile saline. One hundred microliters of the suspensions were inoculated onto thiosulfate-citrate-bile salts-sucrose (TCBS) agar (Difco™, USA) and incubated at 30 °C for 24 hours. Dominant colonies were selected and re-streaked onto new TCBS plates under the same conditions to obtain pure cultures. Bacterial isolates were stored in 20% glycerol at −80 °C for further investigation.

### 2.2. Identification of bacterial isolates by phenotypic characterization

Bacterial isolates were initially identified based on colony morphology, Gram staining, oxidase tests, and pigmentation. Pure colonies were then subjected to biochemical identification using the API® 20E system (bioMérieux, Marcy l’Etoile, France), following the manufacturer’s instructions. API 20E results were entered into the manufacturer’s database for identification (https://apiweb.biomerieux.com). For comparison, previously characterized strains *V. parahaemolyticus* B4 [11], associated with white feces syndrome (*V*_*pWFS*_), and *V. parahaemolyticus* A3 [12], associated with AHPND (*V*_*pAHPND*_), were included in the assay and further analysis. The reference strain *V. parahaemolyticus* ATCC 17802 (American Type Culture Collection) was used for quality control.

### 2.3. Detection of toxin genes

Bacterial genomic DNA extraction from the biochemically identified *V. alginolyticus* (AY7) and *V. parahaemolyticus* (AG1) isolates was performed using the boiling method, as previously described [13]. Briefly, pure colonies were suspended in 100 μL of sterile water and heated in a 95°C heat block for 10 minutes. The tube was then immediately placed on ice for 5 minutes. Next, the sample was centrifuged at 6,000 × g for 5 minutes, and the clear supernatant (containing DNA) was transferred to a new sterile microcentrifuge tube. DNA purity and concentration were measured using a NanoDrop spectrophotometer (Thermo Fisher Scientific, USA), adjusted to 100 ng/μL with distilled water, and stored at −20°C for further PCR use.

The DNA of *Vibrio* strains (AG1 and AY7) was tested for the presence of *pirA* and *pirB* genes, associated with AHPND, and *vhvp-1* and *vhvp-2* genes, linked to TPD, using the primer sets listed in **Table 1**. Each sample was tested using a 25-µL PCR reaction consisting of 1 µL (10 ng DNA template), 12.5 µL of DreamTaq Green PCR Master Mix (2×) (Thermo Scientific™), 0.5 µL of each gene-specific primer, and 10.5 µL of nuclease-free water. Amplification conditions for duplex *pirA/pirB* included an initial denaturation at 94°C for 3 minutes, followed by 35 cycles of 94°C for 30 seconds, 60°C for 30 seconds, and 72°C for 30 seconds, with a final extension at 72°C for 7 minutes (Han et al., 2015). For *vhvp* genes, conditions were 94°C for 4 minutes, followed by 35 cycles of 94°C for 30 seconds, 58°C for 30 seconds, and 72°C for 40 seconds, with a final extension at 72°C for 7 minutes (Qingli, 2020). Following PCR, an aliquot of the products was analyzed using 1.5% agarose gel electrophoresis with 1 × GelRed (Biotium, Fremont, CA, USA). The gel was photographed using the BIORAD Gel Doc XR+ imaging system (Bio-Rad, Hercules, CA, USA). Expected PCR amplicon sizes are provided in **Table 1**.

**Table 1:** The nucleotide sequences of primers used for screening virulence genes of *Vibrio* parahaemolyticus causing AHPND and TPD.

No.	Primer name	Sequence (5’– 3’)	Amplicon size (bp)	Gene screening	References
**1**	VpPirA-F	TGACTATTCTCACGATTGGACTG	284	*pirA*	Han et al. [8]
	VpPirA-R	CACGACTAGCGCCATTGTTA			
**2**	VpPirB-F	TGATGAAGTGATGGGTGCTC	392	*pirB*	
	VpPirB-R	TGTAAGCGCCGTTTAACTCA			
**3**	VpTPD-vhvp-1-F1	GAGGAGAGTGTTGACCGAAATC	362	*vhvp-1*	Liu et al. [7]
	VpTPD-vhvp-1-R1	CTGCGCCAGTAGTAACGATAAG			
**4**	VpTPD-vhvp-2-F1	GGAGTATTGGTGGGCTGAAA	351	*vhvp-2*	
	VpTPD-vhvp-2-R1	GGTAGGCATGGACCGTAAAG			
**5**	VpTPD-vhvp-2-F2	CTAAGCCTTGGCTCCTGAAA	303		
	VpTPD-vhvp-2-R2	CGGTCAGAATATCGGTATCGTAAA			

### 2.4. Pathogenicity tests

To fulfill Koch’s postulates, experimental immersion challenges were conducted using two isolates, *V. alginolyticus* AY7 and *V. parahaemolyticus* AG1, as inoculum in specific pathogen-free (SPF) *P. vannamei* post-larvae. An immersion challenge model was chosen to simulate the natural waterborne infection route typical of aquaculture systems. Two independent trials (Trial 1 and Trial 2) were conducted.

Trial 1 aimed to determine the median lethal concentration (LC_50_) of the isolates in 15-day-old post-larvae (PL15). To prepare the bacterial inoculum, isolates AY7 and AG1 were incubated in Tryptic Soy Broth supplemented with 2% NaCl (TSB+) at 30°C overnight with constant shaking at 150 rpm. Bacteria were centrifuged at 6,000 rpm for 10 minutes at 4°C, washed, and re-suspended in sterile artificial seawater (salinity 25 ppt). Bacterial suspension was adjusted to an OD_600_ of 1.0, corresponding to a concentration of approximately 10^9^ CFU/mL; this conversion was confirmed by conventional plate counting on TCBS agar (100 µL spread plates; ~ 30 °C, 18–24 h). The experimental design consisted of six groups per isolate, receiving immersion challenge doses prepared by serial dilution of the bacterial stock suspension (10^9^ CFU/mL) in artificial seawater to achieve final concentrations ranging from 10^2^ to 10^6^ CFU/mL. Each group contained 20 SPF PL15 in a 4-L jar per concentration, with the temperature maintained at 28 ± 1°C. Each concentration was tested in duplicate, and two negative control groups without added bacteria were included. Mortality was monitored every six hours for 96 hours, and LC_50_ values were calculated according to Hamilton et al. [14].

Given that *V. parahaemolyticus* AG1 was demonstrated to be the primary TPD causative agent based on the presence of *vhvp* toxin genes and high virulence observed in Trial 1, Trial 2 was conducted using only isolate AG1 in larger post-larvae (PL30). The bacterial inoculum was prepared following the same manner as in Trial 1. However, three immersion concentrations were used: 10^2^, 10^3^, and 10^4^ CFU/mL. Ten SPF *P. vannamei* PL30 were challenged per concentration in 90 L tanks. Two replicates were performed, with two negative controls included. Mortality was recorded for seven days.

In both trials, representative moribund shrimp were fixed in Davidson’s alcohol-formalin-acetic acid (AFA) solution for histopathological analysis. Samples from dead shrimp were streaked onto TCBS agar plates for bacterial re-isolation. Additional samples were preserved in 95% ethanol for *vhvp* toxin gene detection by PCR, as described above.

### 2.5. Histopathology

Moribund shrimp from each tank were fixed in Davidson’s AFA solution for 24 hours and then transferred to 70% ethanol. The samples were processed for routine histology, with 5-µm-thick sections cut and stained with hematoxylin and eosin (H&E) following previously established procedures. The H&E-stained slides were scanned using the MoticEasyScan Pro 6 digital microscope scanner (Motic Digital Pathology, Hong Kong). Histopathological changes were examined and photographed using DSAssistant software.

### 2.6. Antibiotic susceptibilities

Upon the pathogenicity test results, the isolate *V. parahaemolyticus* AG1 was selected for antibiotic susceptibility testing using the disk diffusion method [15]. The strain *V*_*pAHPND*_ A3 was also included in the assay and analysis. Fresh colonies from each isolate were suspended in 0.85% saline solution and adjusted to a 0.5 McFarland standard turbidity. The suspension was then uniformly streaked onto Mueller–Hinton agar (HiMedia, India) plates, and antibiotic discs were applied. The test employed commercially available antibiotic discs (Avantor, VWR International, USA). The antibiotics tested included 10 commonly used agents in aquaculture from six classes, with concentrations in μg/disc as follows: penicillin (10 U), ampicillin (10), amoxicillin/clavulanic acid (30), gentamicin (10), streptomycin (10), tetracycline (30), minocycline (30), enrofloxacin (5), levofloxacin (5), ciprofloxacin (5), sulfamethoxazole/trimethoprim (25), and chloramphenicol (30). Plates were incubated at 29 ± 1°C for 18 hours, after which zones of inhibition were measured and interpreted according to Clinical and Laboratory Standards Institute (CLSI) guidelines.

## 3. Results

### 3.1. Bacterial isolation and identification

**Fig 1:**
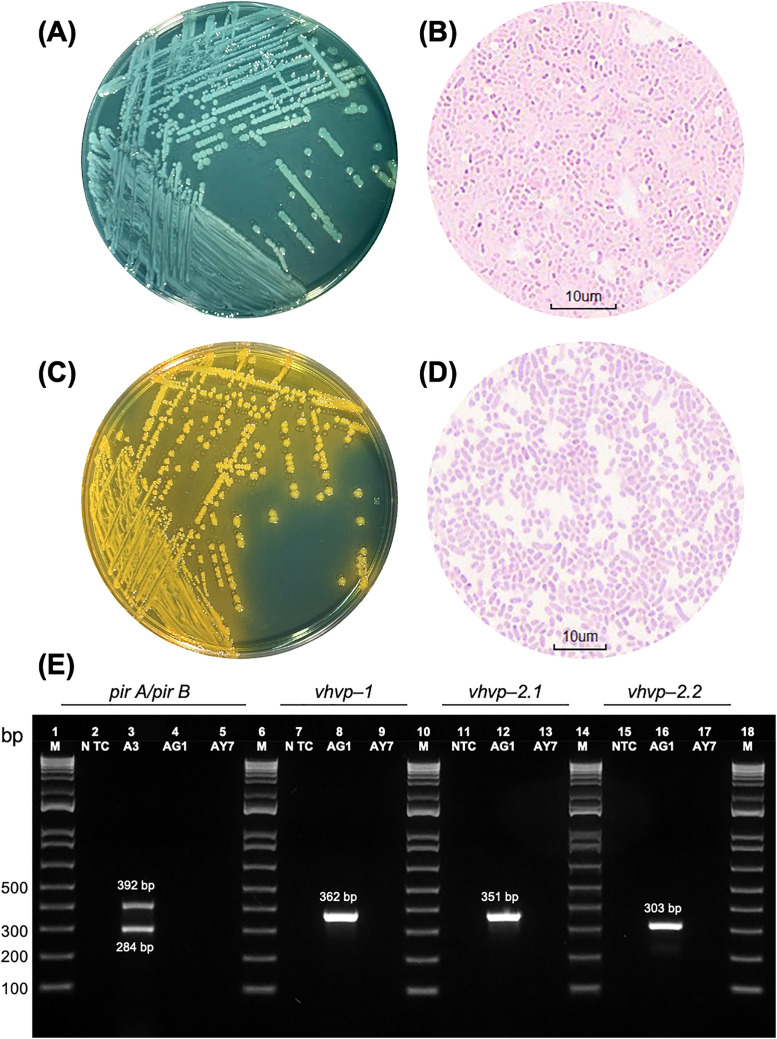
Bacterial isolation and virulence gene screening in bacteria causing TPD. Two colony types were isolated from diseased shrimp on TCBS agar plates: green-colored colonies (A) and yellow-colored colonies (C), designated as AG1 and AY7, respectively. Both isolates exhibited Gram-negative, rod-shaped morphology (B, D). Electrophoretic analysis of toxin gene detection (E). Duplex PCR targeting *pirA* (284 bp) and *pirB* (392 bp) binary toxin genes successfully amplified the target genes in the *V*_*pAHPND*_ A3 reference strain (Lane 3), but no amplification was detected in AG1 and AY7 (Lanes 4, 5). PCR targeting *V*_*pTPD*_-specific virulence genes (*vhvp-1* and *vhvp-2*) was performed using DNA from AG1, yielding amplicons of 362 bp, 351 bp, and 303 bp with the VpTPD-vhvp-1-F1/R1, VpTPD-vhvp-2-F1/R1, and VpTPD-vhvp-2-F2/R2 primer sets (Lanes 8, 12, and 16), respectively. DNA from AY7 did not provide any amplicon when PCR was performed using *V*_*pTPD*_-specific primers (Lanes 9, 13, and 17). M: 1 kb molecular ladder; NTC: no-template control.

Two colony types were isolated from diseased shrimp on TCBS agar plates (**Fig 1A**, C), including green-colored and yellow-colored colonies, designated as AG1 and AY7 isolates, respectively. Both isolates were Gram-negative, rod-shaped morphology (**Fig 1B**, D). After biochemical characterization and identification using the API® 20E system, the isolates were confirmed as *V. parahaemolyticus* (AG1) and *V. alginolyticus* (AY7) (**Table 2**). Notably, the identification was further validated by genotypic characterization of *V. parahaemolyticus* AG1, which was identified as the primary TPD causative agent. The genomic data is available in the Sequence Read Archive (SRA) database (PRJNA1193633) and will be reported in a subsequent study (*Mai et al., unpublished*).

**Table 2:** Phenotypic identification and biochemical characterization of AG1 and AY7 isolates of *Vibrio* spp*. Vibrio parahaemolyticus* A3 (*V*_*pAHPND*_) and *V. parahaemolyticus* B4 (*V*_*pWFS*_) were included for comparison, while the reference strain *V. parahaemolyticus* ATCC 17802 was used as quality control.

Characteristic	AY7	AG1	*V. parahaemolyticus* A3 (*V*_*AHPND*_)	*V. parahaemolyticus* B4 (*V*_*WFS*_)	*V. parahaemolyticus* ATCC 17802
Growth on TCBS	Yellow	Green	Green	Green	Green
Gram staining	−	−	−	−	−
Cell morphology	Rods	Rods	Rods	Rods	Rods
β-galactosidase (ONPG)	−	−	−	−	−
Arginine dihydrolase (ADH)	−	−	−	−	−
Lysine decarboxylase (LDC)	+	+	+	+	+
Ornithine decarboxylase (ODC)	−	+	+	+	+
Citrate utilization (CIT)	−	−	−	−	−
Production of H2S (H2S)	−	−	−	−	−
Urease production (URE)	−	−	−	−	−
Tryptophan deaminase (TDA)	+	−	+	+	+
Indole production (IND)	+	+	+	+	+
Voges-Proskauer (VP)	−	−	−	−	−
Gelatin hydrolysis (GEL)	−	+	+	+	+
Glucose fermentation (GLU)	+	+	+	+	+
Mannitol fermentation (MAN)	+	+	+	+	+
Inositol fermentation (INO)	−	−	−	−	−
Sorbitol fermentation (SOR)	−	−	−	−	−
Rhamnose fermentation (RHA)	−	−	−	−	−
Sucrose fermentation (SAC)	+	−	−	−	−
Melibiose fermentation (MEL)	−	−	−	−	−
Amygdalin fermentation (AMY)	+	−	−	+	−
Arabinose fermentation (ARA)	−	−	−	−	+
Cytochrome oxidase (OX)	+	+	+	+	+
API 20E profile (% identification)	*V. alginolyticus* (99.2)	*V. parahaemolyticus* (89.7)	*V. parahaemolyticus* (89.7)	*V. parahaemolyticus* (83.8)	*V. parahaemolyticus* (99.9)

+, positive; − , negative

### 3.2. Virulence gene screening

Duplex PCR detection for the *pirA* (284 bp) and *pirB* (392 bp) binary toxin genes was conducted using DNA from isolates AG1 and AY7, but no amplification was detected. In contrast, the *V*_*pAHPND*_ A3 reference strain successfully amplified the target genes (**Fig 1E**). PCR targeting *V*_*pTPD*_-specific virulence genes (*vhvp-1* and *vhvp-2*) was performed using DNA from isolate AG1, yielding 362 bp, 351 bp, and 303 bp amplicons with the VpTPD-vhvp-1-F1/R1, VpTPD-vhvp-2-F1/R1, and VpTPD-vhvp-2-F2/R2 primer sets, respectively. However, DNA from strain AY7 failed to amplify using these *V*_*pTPD*_-specific primer sets (**Fig 1E**). These findings indicate that *V. parahaemolyticus* AG1 lacks the *pirA/pirB* binary toxin genes but harbors *vhvp-1* and *vhvp-2*, whereas *V. alginolyticus* AY7 lacks both *V*_*pAHPND*_ – and *V*_*pTPD*_ -associated virulence genes.

### 3.3. Pathogenicity of the bacterial isolates

**Fig 2:**
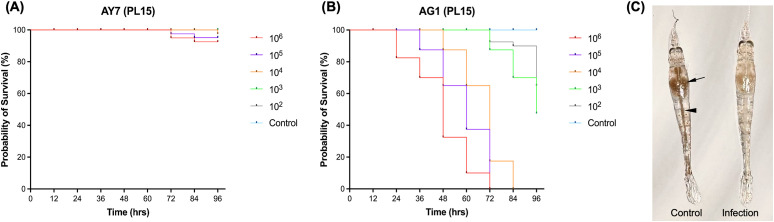
Pathogenicity of *V. alginolyticus* AY7 (A) and *V. parahaemolyticus* AG1 (B) in *P. vannamei* PL15 (n = 40 per dose) over a 96-hour immersion challenge. Shrimp exposed to AY7 at concentrations up to 10⁶ CFU/mL exhibited minimal mortality (7.5%) without gross pathological signs. In contrast, AG1 demonstrated dose-dependent pathogenicity, with 100% mortality at 10⁶ and 10⁵ CFU/mL within 72 hours. Intermediate concentrations (10^4^ and 10^3^ CFU/mL) resulted in a gradual decline in survival, while the 10^2^ CFU/mL group showed moderate mortality (35%). The LC_50_ for AG1 was calculated as 8.51 × 10^2^ CFU/mL at 96 hours. A healthy post-larva shows a darkened hepatopancreas (arrow) and a distinct intestine (arrowhead), whereas infected post-larvae exhibit a pale or colorless hepatopancreas and an empty digestive tract resulting in a transparent or translucent body (C).

For Trial 1, Kaplan-Meier survival analysis demonstrated the pathogenicity of *V. alginolyticus* AY7 and *V. parahaemolyticus* AG1 in *P. vannamei* post-larvae (PL15) over a 96-hour immersion challenge (**Fig 2A**, B). Shrimp exposed to AY7 at concentrations up to 10⁶ CFU/mL exhibited minimal mortality (7.5%) without gross pathological signs, with survival rates remaining above 95%–100% across all treatment groups, indicating low virulence. In contrast, AG1 induced a dose-dependent pathogenic effect, with significant mortality observed at higher concentrations. Shrimp exposed to 10^6^ and 10^5^ CFU/mL experienced nearly 100% mortality within 72 hours. Intermediate concentrations (10^4^ and 10^3^ CFU/mL) led to a gradual decline in survival, while the 10^2^ CFU/mL group exhibited moderate mortality (35%). No mortality was recorded in the control groups. Consistent with the mortality data, green-colored colonies on TCBS agar were re-isolated from representative dead shrimp in the AG1 treatment groups, confirming Koch’s postulates. Additionally, PCR analysis detected *vhvp-1* and *vhvp-2* genes in DNA extracted from both bacterial colonies and infected shrimp tissue (S1A Fig). The LC_50_ for AG1 was calculated as 8.51 × 10^2^ CFU/mL at 96 hours, further supporting its high virulence as the primary causative agent of TPD. Infected post-larvae exhibited a pale or colorless hepatopancreas and an empty digestive tract, resulting in transparent and translucent body, clinical signs that are consistent with naturally infected shrimp with TPD (**Fig 2C**).

**Fig 3:**
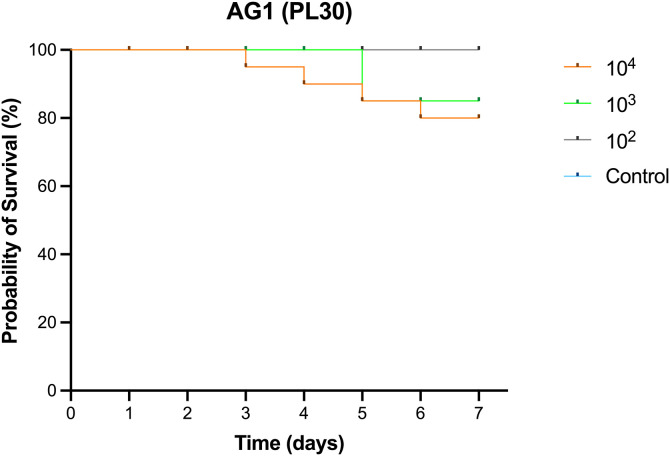
Kaplan-Meier survival analysis of *P. vannamei* PL30 experimentally challenged with *V. parahaemolyticus* AG1 (n = 20 per dose) over a 7-day immersion challenge. Low pathogenicity was observed, with the highest mortality recorded at 20% in the 10⁴ CFU/mL group, followed by 15% in the 10^3^ CFU/mL group.

For Trial 2, Kaplan-Meier survival analysis evaluated the pathogenicity of *V. parahaemolyticus* AG1 in larger post-larvae (PL30) over a 7-day immersion challenge (**Fig 3**). Low pathogenicity was observed, with shrimp exposed to 10⁴ CFU/mL experiencing the highest mortality, reaching only 20%. Unlike PL15 shrimp in Trial 1, infected PL30 shrimp exhibited non-specific clinical signs, with no distinct gross pathology, such as a pale hepatopancreas or empty digestive tract.

### 3.4. Histopathology finding

**Fig 4:**
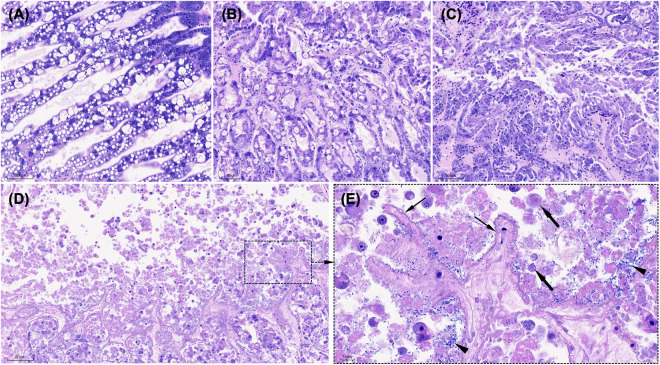
Histopathological analysis of the hepatopancreas in *P. vannamei* PL15 experimentally challenged with *V. parahaemolyticus* AG1. The control group displayed normal histological architecture, with intact hepatopancreas tubule structure and abundant lipid deposits (A). In the early phase of infection, hepatopancreatic tubules exhibited contraction with mild necrosis (B). By the middle phase of infection, intestinal epithelial cells detached, and sloughing lesions intensified (C). In the later phase of infection, severe structural degradation of hepatopancreas tubule was evident (D), with the complete loss of elliptical and circular luminal structures and extensive tubular necrosis (thin arrows in E), severe sloughing of epithelial cells and tissue debris (arrows), accompanied by massive rod-shaped bacterial colonization (arrowheads). Slides were stained with Mayer–Bennet hematoxylin and eosin-phloxine (H&E). Scale bars are included in the images (80 μm in panels A–D, except 10 μm in panel E).

The hepatopancreas of PL15 post-larvae from the uninfected group exhibited normal histological architecture, with intact tubule structure and abundant lipid deposits (**Fig 4A**). In contrast, histopathological analysis of *P. vannamei* infected with *V*_*pTPD*_ AG1 showed a consistent pattern of disease progression across all exposure groups. In the early phase of infection (**Fig 4B**), hepatopancreatic tubules contracted with mild necrosis. As infection progresses (**Fig 4C**), tubule epithelial cells detached, and progressively sloughed into the tubule lumen, although bacterial accumulation was not frequently observed instead occurred occasionally. In the later phase of infection (**Fig 4D**), the hepatopancreas exhibited severe structural degradation, characterized by the complete loss of elliptical and circular luminal structures and extensive necrosis of the tubular (**Fig 4E**). Severe sloughing of epithelial cells and tissue debris was observed, accompanied by massive bacterial colonization. Melanosis was absent in all infected larvae throughout the 96-hour infection period, confirming the rapid and severe progression of TPD infection.

**Fig 5:**
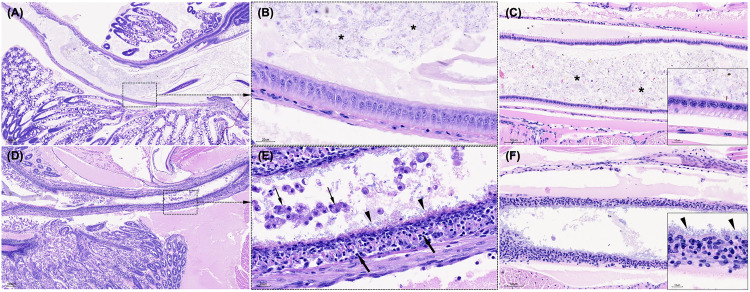
Histopathological analysis of the intestine in *P. vannamei* PL15 artificially infected with *V. parahaemolyticus* AG1The healthy animals (i.e., control treatment) displayed a well-defined peritrophic membrane (A, midgut; C, hindgut) surrounding digested food (asterisks in B and C) and a structured epithelial cell arrangement (B and box in C). In contrast, infected shrimp intestines lacked gut content (D, midgut; F, hindgut), extensive sloughing of epithelial cells (thin arrows in E) and massive rod-shaped bacterial invasion into the intestinal epithelium (arrowheads in D, F). Hemocytic enteritis was observed, characterized by mucosal epithelium loss, intense inflammation, and a thick hemocyte layer (arrows in E and box in F). Slides were stained with Mayer–Bennet hematoxylin and eosin-phloxine (H&E). Scale bars are included in the images (100 μm in panels A and D, 50 μm in panels C and F, and 20 μm in panels B and E).

**Fig 6:**
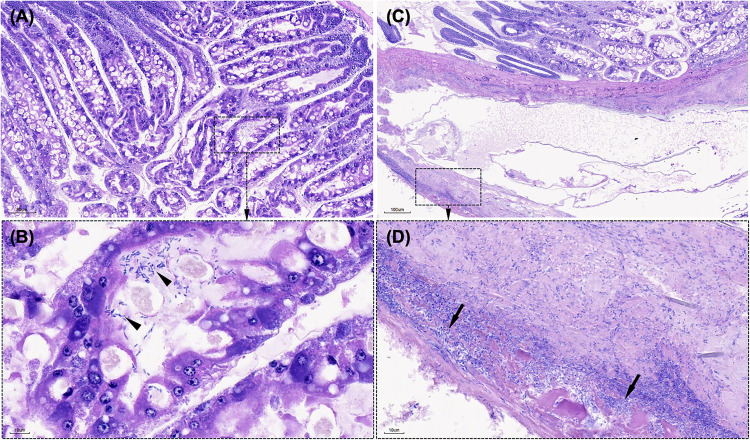
Histopathology of hepatopancreas and intestine in *Penaeus vannamei* PL30 experimentally challenged with *Vibrio parahaemolyticus* AG1. Unlike *P. vannamei* PL15 (see Fig 5), histopathological lesions were less severe with a limited progression of infection. The hepatopancreas exhibited early-stage infection (A), characterized by tubule contraction and minimal bacterial accumulation (arrowheads in B). A heavy bacterial load in the intestine (C) and massive bacterial invasion into the intestinal epithelium (arrows in D) were observed, suggest that mortality may have resulted from the acute effects of a high bacterial load. Slides were stained with Mayer–Bennet hematoxylin and eosin-phloxine (H&E). Scale bars are included in the images (100 μm in panel C, 80 μm in panel A, and 10 μm in panels B and D).

In the intestine, the control groups exhibited a well-defined peritrophic membrane surrounding digested food and a structured epithelial cell arrangement (**Fig 5A**–C). In contrast, PL15 shrimp infected with *V*_*pTPD*_ AG1 had intestines devoid of gut content (**Fig 5D**–F), with extensive sloughing of epithelial cells, and massive bacterial invasion into the intestinal epithelium. Hemocytic enteritis, a typical histopathological change was observed and characterized by mucosal epithelium loss, intense inflammation, and a thick hemocyte layer. In the PL30 shrimp group, mortality was very low, and histopathological changes observed in these shrimp were less severe, with limited infection progression. The hepatopancreas primarily exhibited early-stage infection (**Fig 6A**), characterized by contraction of the tubules and minimal bacterial accumulation (**Fig 6B**). However, a heavy bacterial load in the intestine (**Fig 6C**) and massive bacterial invasion into the intestinal epithelium were observed (**Fig 6D**).

Histopathological examination of PL15 shrimp infected with *V. alginolyticus* AY7 revealed intact epithelial cell arrangements in the hepatopancreatic tubules and intestine, with digested food present and no observable pathological changes (S1B Fig), consistent with the negative control. Additionally, the surviving PL30 shrimp at the end of the experiment, as well as those in the control group, showed no pathological manifestations of infection (S1C Fig).

### 3.5. Antibiotic susceptibility pattern

The antimicrobial resistance profiles of *V*_*pTPD*_ AG1 and *V*_*pAHPND*_ A3 are shown in **Table 3**. Both strains exhibited an identical resistance pattern, showing resistance to penicillin and ampicillin, which belong to the β-lactam class. Additionally, both strains were susceptible to the remaining 10 antibiotic agents, including amoxicillin/clavulanic acid, gentamicin, streptomycin, tetracycline, minocycline, enrofloxacin, levofloxacin, ciprofloxacin, sulfamethoxazole/trimethoprim, and chloramphenicol.

**Table 3:** Antibiotic susceptibility profiles of *V. parahaemolyticus* AG1 (*V*_*pTPD*_) and *V. parahaemolyticus* A3 (*V*_*pAHPND*_).

Antimicrobial classes	Antimicrobial agents	Concentration (µg/disc)	Resistance profile			
			*V. parahaemolyticus AG1 (V*_*pTPD*_ *)*		*V. parahaemolyticus A3 (V*_*pAHPND*_ *)*	
			Zone of inhibition (Diameter, mm)	Interpretation	Zone of inhibition (Diameter, mm)	Interpretation
**Penicillins** **(β-lactams)**	Penicillin (PEN)	10 units	0	R	0	R
	Ampicillin (AMP)	10	0	R	0	R
	Amoxicillin/Clavulanic acid (AMX)	30	25	S	23	S
**Aminoglycosides**	Gentamicin (GEN)	10	21.5	S	19.5	S
	Streptomycin (STR)	10	22.5	S	19.5	S
**Tetracyclines**	Tetracycline (TE)	30	30	S	27	S
	Minocycline (MI)	30	26.5	S	24.5	S
**Fluoroquinolones**	Enrofloxacin (ENO)	5	25.5	S	24	S
	Levofloxacin (LE)	5	30	S	23.5	S
	Ciprofloxacin (CIP)	5	29	S	26	S
**Sulfonamides**	Sulfamethoxazole /Trimethoprim (SXT)	25	31	S	26.5	S
**Amphenicols**	Chloramphenicol (CHL)	30	32.5	S	30	S
**Summary**			R (2), I (0), S (10)		R (2), I (0), S (10)	

R, resistant; S, sensitive; I, intermediate.

*Zone of inhibition values represent the mean of two independent replicates, with a SD of less than 1 mm.*

## 4. Discussion

In early 2020, a highly lethal disease, translucent post-larvae disease (TPD), emerged in *P. vannamei* post-larvae (PL6–12) in shrimp hatcheries across Guangdong and Fujian Provinces, China, causing up to 100% mortality within 72 hours [4]. The disease rapidly spread across southern China, forcing the closure of 70–80% of hatcheries and nurseries in major shrimp farming provinces. Highly virulent strains of *V. parahaemolyticus* (*V*_*pTPD*_) was identified as the causative agent, leading to severe economic losses and threatening the sustainability of China’s shrimp industry [4,6]. Here, we report the first confirmed isolation, identification, and experimental reproduction of *V*_*pTPD*_, outside China, with re-isolation of the challenge strain and gene-level confirmation, thereby fulfilling Koch’s postulates. Within the study period, this investigation represented the first laboratory-confirmed and isolated *V*_*pTPD*_ case in our collaborator network outside China. Although similar clinical signs were anecdotally reported at other facilities in the region, suitable diagnostic submissions for bacterial isolation were not received; therefore, additional cases could not be confirmed. Detection of *V*_*pTPD*_ AG1 in a new geographical region underscores the risk of transboundary spread through shrimp trade and live animal transfers, emphasizing the need for heightened biosecurity measures and targeted surveillance.

Experimental challenges demonstrated that *V*_*pTPD*_ AG1 exhibits high virulence in early-stage shrimp (PL15), with an LC_50_ of 8.51 × 10^2^ CFU/mL at 96 hours, consistent with previous reports showing that *V*_*pTPD*_ strains cause severe mortality *Penaeus vannamei* [4,6]. While LC_50_ data for other *V*_*pTPD*_ strains are unavailable, Yang et al. [6] reported that the median lethal time (LT_50_) for shrimp infected with *V*_*pTPD*_ vp-HL-202005 at 1 × 10^3^ cells/mL was 59 hours, comparable to the 56-hour LT_50_ observed in shrimp infected with *V*_*pAHPND*_ vp-pir-201806 at 1 × 10⁶ cells/mL. This indicating that *V*_*pTPD*_ could achieve achieve comparable lethality at ~10^3^-fold lower doses, implying greater virulence than the *V*_*pAHPND*_ strain. Similarly, Zou et al. [4] reported an LT_50_ of 38.6 hours in shrimp challenged with *V*_*pTPD*_ JS20200428004–2 at 1.83 × 10⁴ cells/mL, while infection with *V*_*pTPD*_ vp-HL-202005 at 1 × 10⁴ cells/mL resulted in an LT_50_ of 38 hours, further demonstrating the high pathogenicity of *V*_*pTPD*_ strains. In line with this, and relative to published *V*_*pAHPND*_ strains, *V*_*pTPD*_ appears to have markedly lower LC₅₀ and shorter LT₅₀ in PL, suggesting a substantially higher per-exposure risk at bacterial loads commonly encountered in hatchery environments. This elevates the importance of: (i) stringent control of incoming water and live inputs (broodstock, *Artemia*, algal starters); (ii) early and frequent screening (qPCR or culture) during PL1–PL10; (iii) reducing effective exposure dose through rigorous sanitation measures (e.g., chlorination, ozonation, UV treatment) and maintaining a one-way biosecurity flow; and (iv) rapid removal of heavily affected cohorts to limit tank-to-tank transmission. Because TPD primarily affects very young stages, even brief lapses in hygiene can result in clinically significant exposure; consequently, proactive prophylaxis and surveillance are likely to be more cost-effective and impactful than reactive interventions. Interestingly, mortality in PL30 shrimp was significantly lower (i.e., 20% at 10⁴ CFU/mL over 7 days), indicating size-dependent resistance. This aligns with previous studies indicating that the disease primarily affects post-larval stages (P5–P12) of *P.vannamei*, characterized by short outbreak durations and high mortality rates [4,6]. This reduced susceptibility in older PLs is likely due to enhanced innate immunity, including hemocyte activation, antimicrobial peptide production, and improved integrity of epithelial cells in the hepatopancreatic tubule and midgut epithelium which may limit bacterial invasion and systemic spread. Histopathological analysis further supports this, revealing limited bacterial accumulation in the hepatopancreas and only early-stage lesions in PL30 shrimp. The low mortality and histopathological changes observed in these shrimp, characterized by massive bacterial invasion of the intestinal epithelium, suggest that their deaths may have resulted from the acute effects of a high bacterial load. A mature gut microbiome may also contribute to resistance by outcompeting pathogenic *Vibrio* strains [10]. Further research is needed to elucidate the immunological and microbiome-mediated mechanisms underlying this resistance and to develop targeted disease prevention strategies in shrimp aquaculture.

Interestingly, *V. alginolyticus* AY7, despite being isolated from diseased shrimp, was non-virulent in experimental challenges, with minimal mortality (7.5%) even at 10⁶ CFU/mL and no clinical signs of disease. This suggests AY7 strain is an opportunistic *V. alginolyticus* strain rather than a primary pathogen causing TPD. Similar cases have been reported where non-virulent *Vibrio* species were co-isolated with pathogenic strains, likely exploiting host immunosuppression or environmental imbalances [16,17]. The absence of *vhvp-1* and *vhvp-2* in *V. alginolyticus* AY7 supports this, as these genes are critical virulence determinants of TPD [5]. Histopathological analysis further confirms this, showing intact hepatopancreatic and intestinal structures, with no observable pathological changes, consistent with negative controls. These findings suggest that *V. alginolyticus* AY7 does not independently cause disease but may proliferate opportunistically in immunocompromised shrimp, warranting further investigation into its ecological role in disease outbreaks.

Histopathological examination of *V*_*pTPD*_ AG1-infected shrimp revealed progressive hepatopancreatic degradation, characterized by tubular necrosis, loss of luminal structures, and extensive bacterial colonization that are hallmarks of severe *Vibrio*-induced pathology [18–21]. These findings also align with reports of TPD outbreaks in China [4–7,10]. While *V*_*pTPD*_ infection in the *P. vannamei* post-larvae caused histopathological changes similar to AHPND to some degree, including epithelial cell sloughing and tubule destruction [8,12], pathological manifestation in the intestine distinguished *V*_*pTPD*_ AG1 from AHPND. Unlike AHPND, where *pirA/pirB* toxins induce widespread enterocyte sloughing and midgut epithelial collapse, *V*_*pTPD*_ AG1-infected shrimp exhibited hemocytic enteritis, gut epithelial loss, and bacterial invasion of the intestinal mucosa, potentially indicating an immune response with hemocyte recruitment to infection sites. Melanosis was notably absent, suggesting that TPD progresses too rapidly for an effective melanization response, a key immune defense against chronic bacterial infections [22]. The detection of *vhvp-2* gene in the *V*_*pTPD*_ AG1 strain suggests a potential role in gut pathology and systemic bacterial dissemination. While previous research suggests *vhvp-2* may play a role in *V. parahaemolyticus* adhesion and invasion [7], its involvement in quorum-sensing mechanisms remains unclear and requires further investigation.

Antibiotic susceptibility testing showed that *V*_*pTPD*_ AG1 was resistant to β-lactams (penicillin and ampicillin), raising concern due to the widespread use of β-lactams in shrimp disease management and their broader implications for antimicrobial resistance in aquaculture [23]. However, *V*_*pTPD*_ AG1 remained sensitive to fluoroquinolones, aminoglycosides, tetracyclines, sulfonamides, and phenicols, indicating that alternative treatment options are available. Despite these alternatives, reliance on antibiotics is not sustainable, as overuse can accelerate resistance development and disrupt microbial homeostasis in aquaculture environments. The growing demand for antibiotic-free shrimp production has led to increased reliance on biosecurity measures, such as early detection and disinfection [5,24] to control TPD outbreaks. Identifying key virulence factors of *V*_*pTPD*_ is crucial for developing diagnostic tools and preventive strategies. The strong association of *vhvp-1* and *vhvp-2* with *V*_*pTPD*_ outbreaks reinforces their role in pathogenicity, underscoring the need for molecular diagnosis in TPD surveillance [5].

A major concern is the genetic plasticity of *V. parahaemolyticus*, which enables horizontal gene transfer of virulence factors, facilitating the emergence of novel pathogenic strains [25]. The detection of *vhvps* in the *V*_*pTPD*_ AG1 strain isolated outside China raises concerns about potential recombination with other *Vibrio* species, which could lead to hypervirulent variants. Previous studies have documented *V. parahaemolyticus* acquiring new virulence determinants, enhancing environmental adaptability and host range [17,26]. The emergence of *V*_*pTPD*_ outside China suggests that climate variability, intensified aquaculture practices, and global shrimp trade may accelerate disease spread. Continuous genomic surveillance of *V*_*pTPD*_ is essential to track virulence gene variations, detect pathogenicity shifts, and prevent future outbreaks. These efforts will be critical for early threat detection and the development of effective disease control strategies in shrimp aquaculture.

In conclusion, this study confirms the presence of *V*_*pTPD*_ outside China, characterizes its pathogenicity, and highlights a potential risk of global transmission. While early-stage post-larvae (PL15) of *P. vannamei* exhibited high susceptibility, larger post larvae (PL30) showed increased resistance, suggesting a size-dependent defense mechanism. Histopathological analysis revealed progressive hepatopancreatic degradation and intestinal pathology with hemocytic enteritis that are distinct from pathological manifestations associated with AHPND. The detection of *vhvps* in *V*_*pTPD*_ AG1 reinforces their role in pathogenicity and, along with β-lactam resistance, raises concerns for disease management. These findings underscore the urgent need for TPD surveillance, enhanced biosecurity, and alternative disease control strategies to mitigate the spread of *V*_*pTPD*_ in shrimp aquaculture.

## Supporting information

S1 Fig (A) PCR analysis detected *vhvp-1* and *vhvp-2* genes in DNA extracted from bacterial colonies and infected shrimp tissue in the *V. parahaemolyticus* AG1 infection group. M: 1 kb molecular ladder; AG1: DNA from AG1 pure colonies; NTC: no-template control; C1, C2, C3: DNA from representative bacterial colonies isolated from dead experimental shrimp; S1, S2, S3: DNA from representative dead experimental shrimp. (B) Histopathological examination of PL15 shrimp infected with *V. alginolyticus* AY7 revealed intact epithelial cell arrangements in the hepatopancreatic tubules and intestine, with digested food present and no observable pathological changes, consistent with the negative control. (C) The surviving PL30 shrimp at the end of the experiment in the *V. parahaemolyticus* AG1 infection group showed no pathological manifestations of infection. Scale bars are included in the images (100 μm in panels B and C).
(TIF)

S1 File Unprocessed figures. (ZIP)
