# Supplementary figures and images for: Isolation, characterization, and pathogenicity of a Vibrio parahaemolyticus strain causing translucent post-larvae disease in Penaeus vannamei outside China
Source: PLoS One. 2025 Sep 15;20(9):e0331862. doi: 10.1371/journal.pone.0331862 (PMC12435696; doi:10.1371/journal.pone.0331862)

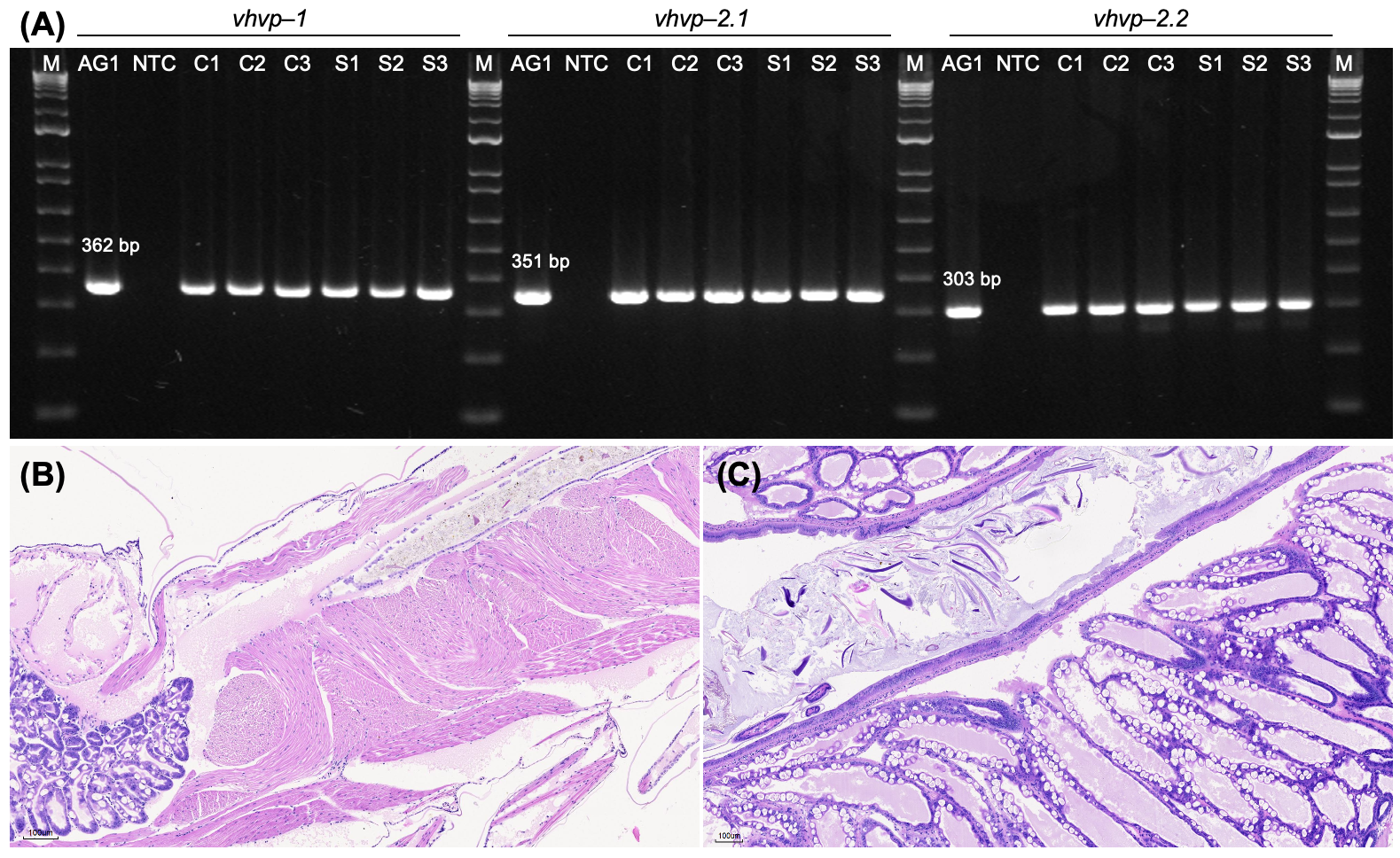

Supplement: S1 Fig — M: 1 kb molecular ladder; AG1: DNA from AG1 pure colonies; NTC: no-template control; C1, C2, C3: DNA from representative bacterial colonies isolated from dead experimental shrimp; S1, S2, S3: DNA from representative dead experimental shrimp. (B) Histopathological examination of PL15 shrimp infected with V. alginolyticus AY7 revealed intact epithelial cell arrangements in the hepatopancreatic tubules and intestine, with digested food present and no observable pathological changes, consistent with the negative control. (C) The surviving PL30 shrimp at the end of the experiment in the V. parahaemolyticus AG1 infection group showed no pathological manifestations of infection. Scale bars are included in the images (100 μm in panels B and C). (TIF) [file pone.0331862.s001.tif]

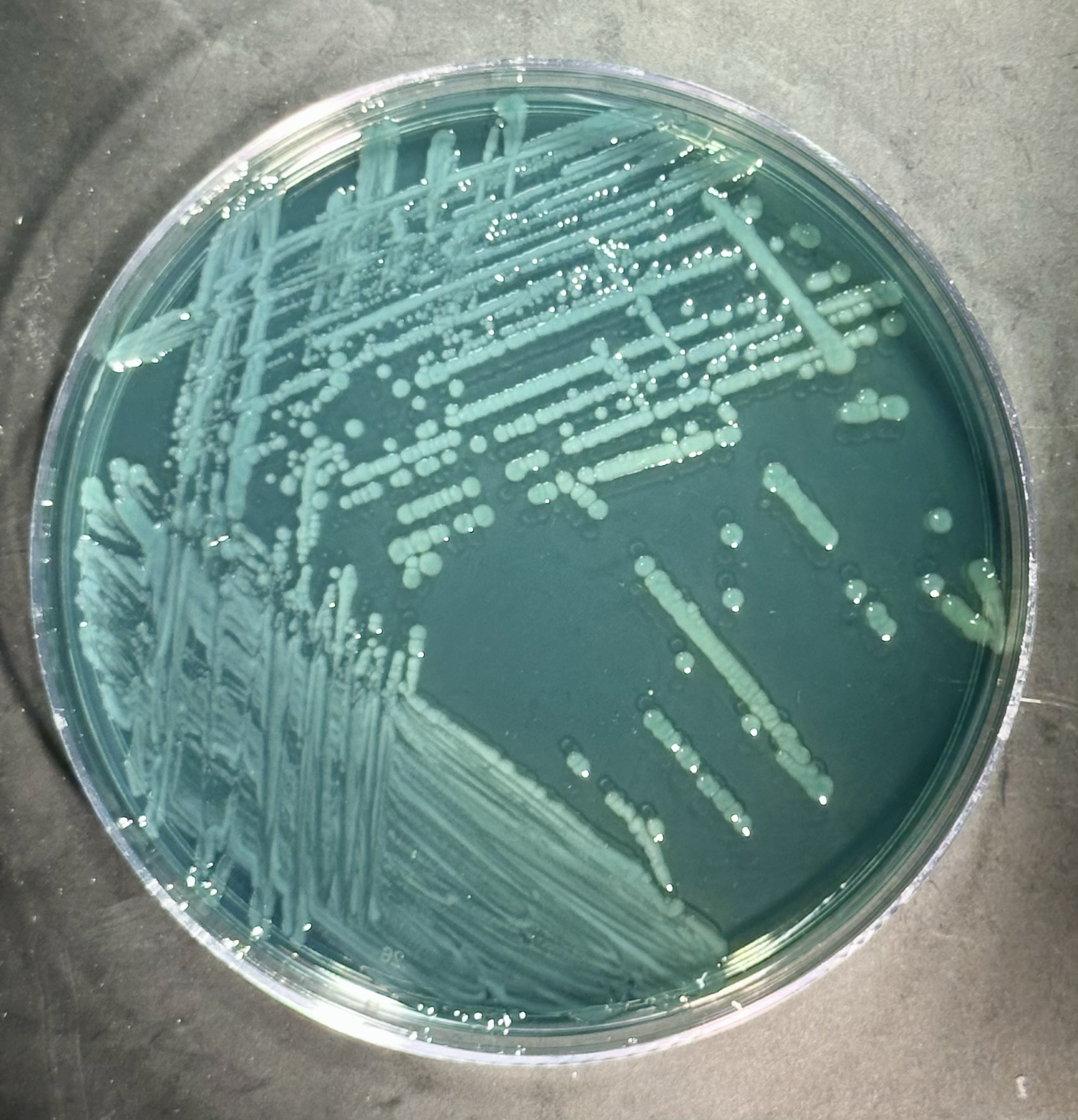

Supplement: S1 File — (ZIP) [file pone.0331862.s002.zip › S1 File/1A.tif]

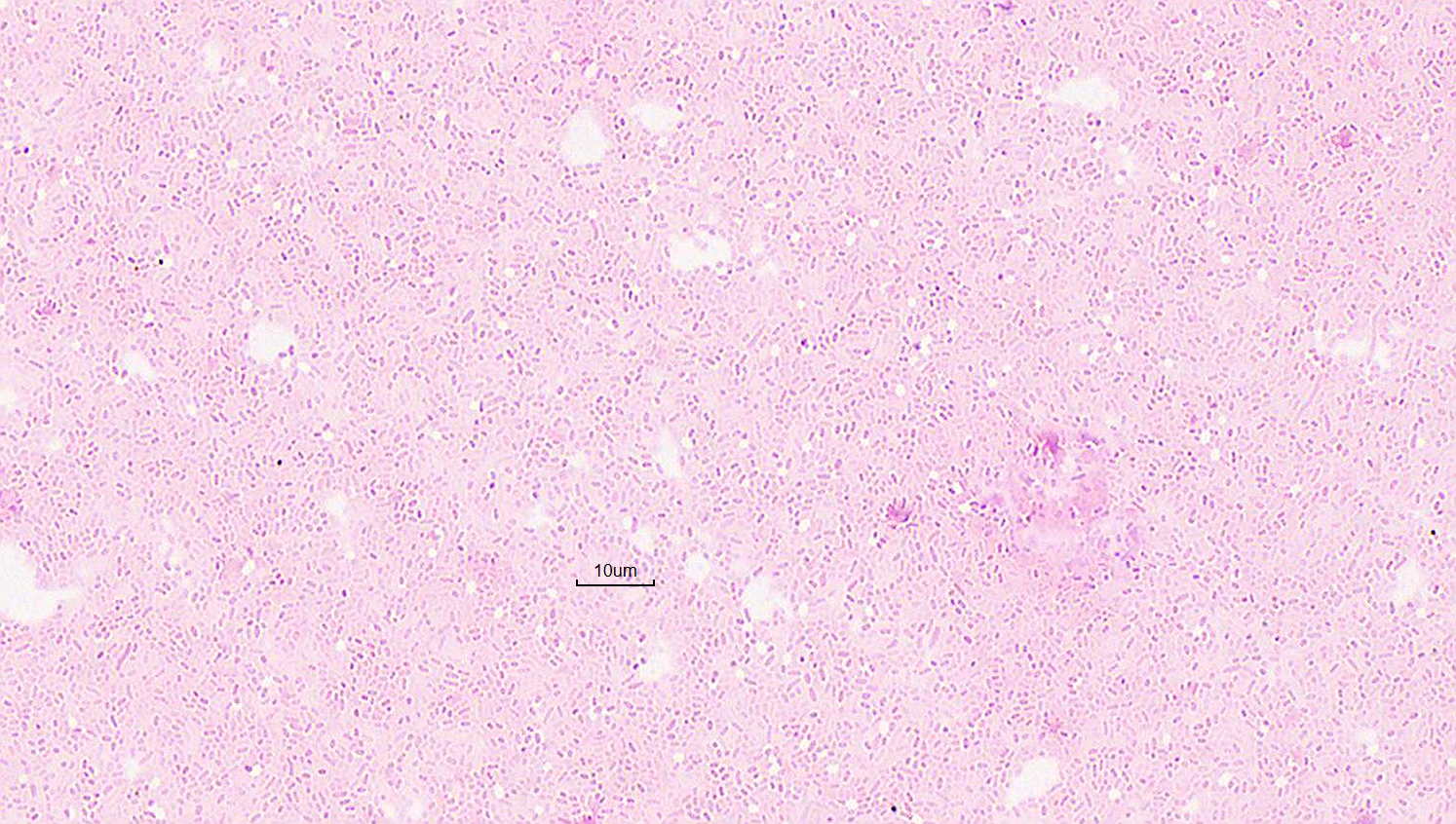

Supplement: S1 File — (ZIP) [file pone.0331862.s002.zip › S1 File/1B.tif]

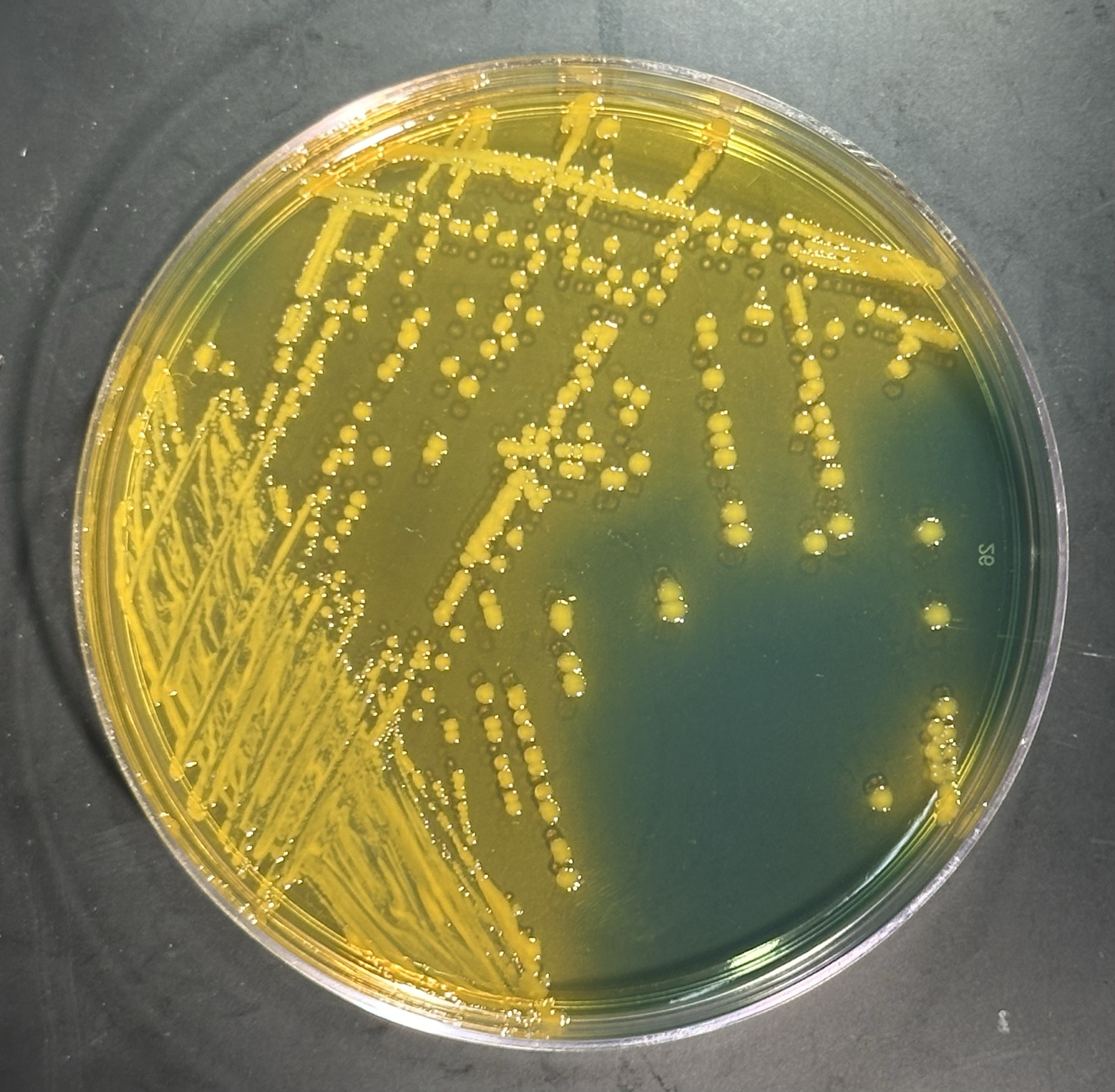

Supplement: S1 File — (ZIP) [file pone.0331862.s002.zip › S1 File/1C.tif]

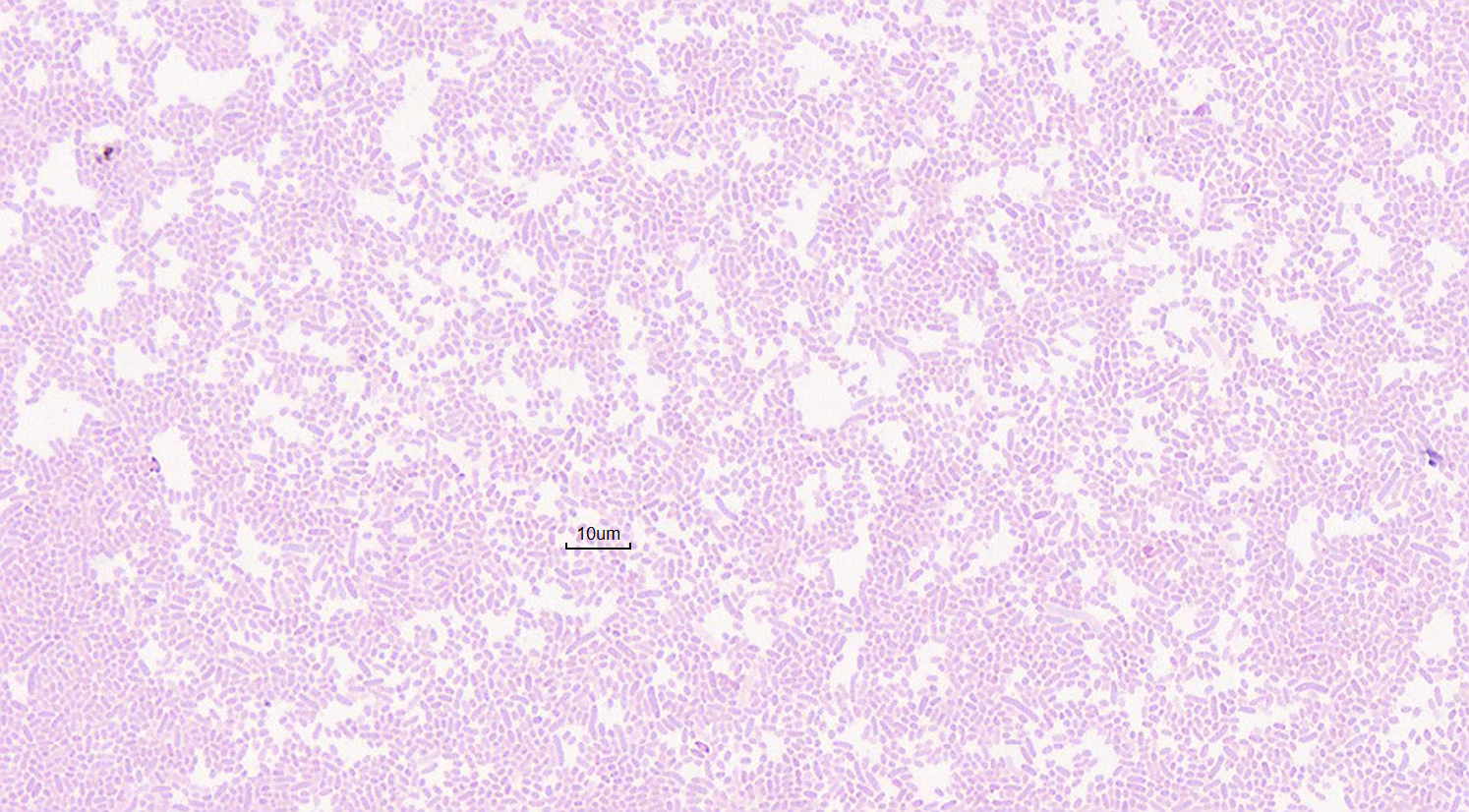

Supplement: S1 File — (ZIP) [file pone.0331862.s002.zip › S1 File/1D.tif]

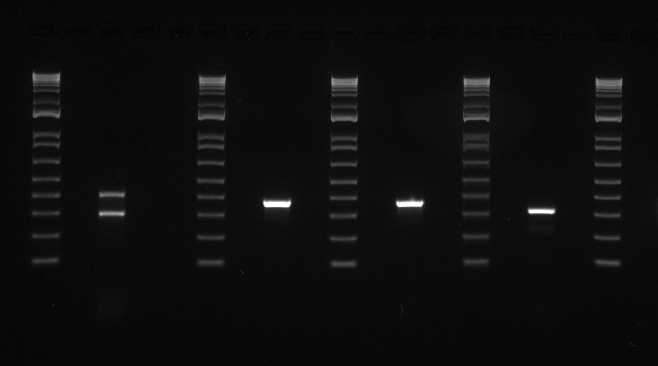

Supplement: S1 File — (ZIP) [file pone.0331862.s002.zip › S1 File/1E.tif]

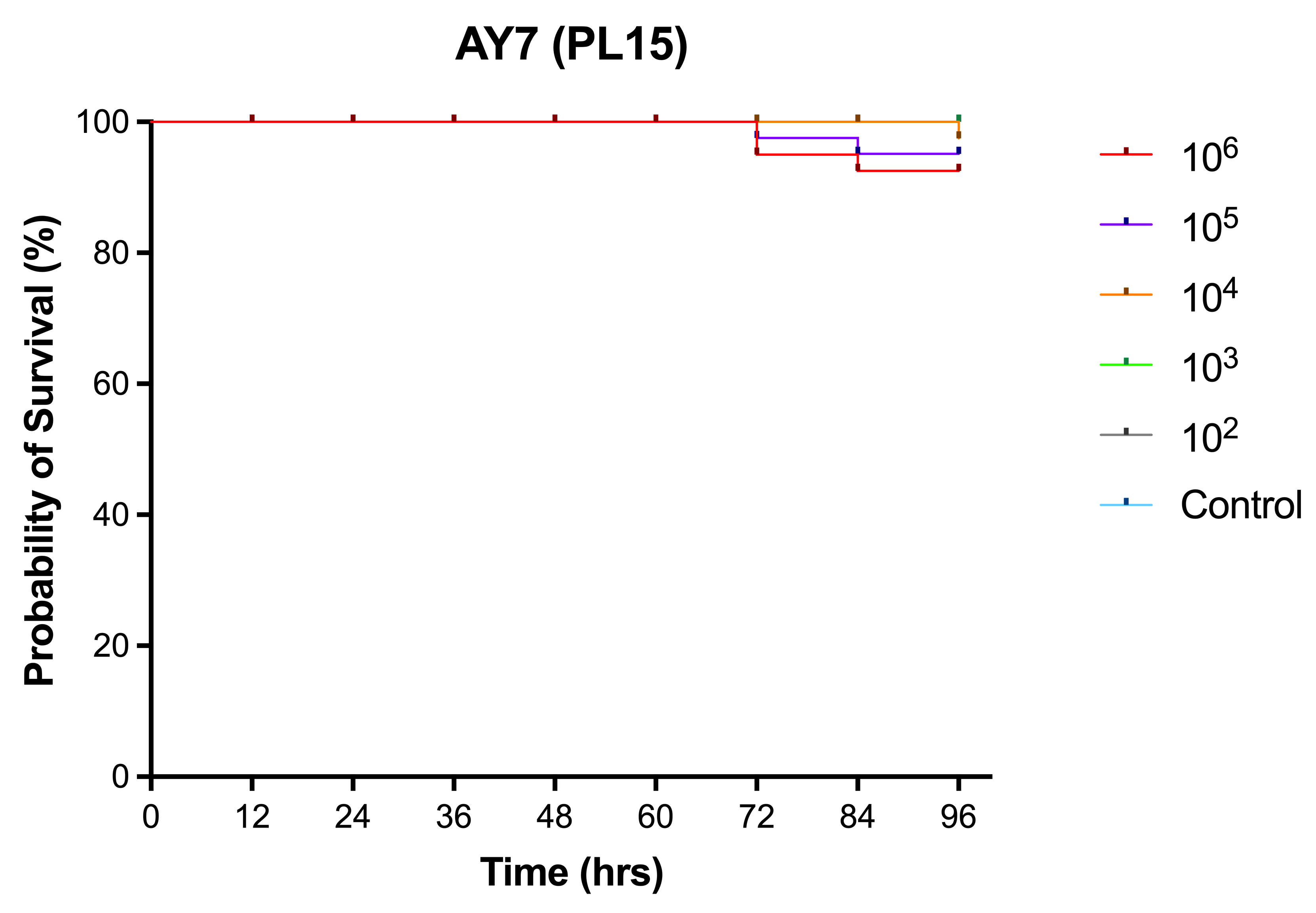

Supplement: S1 File — (ZIP) [file pone.0331862.s002.zip › S1 File/2A.tif]

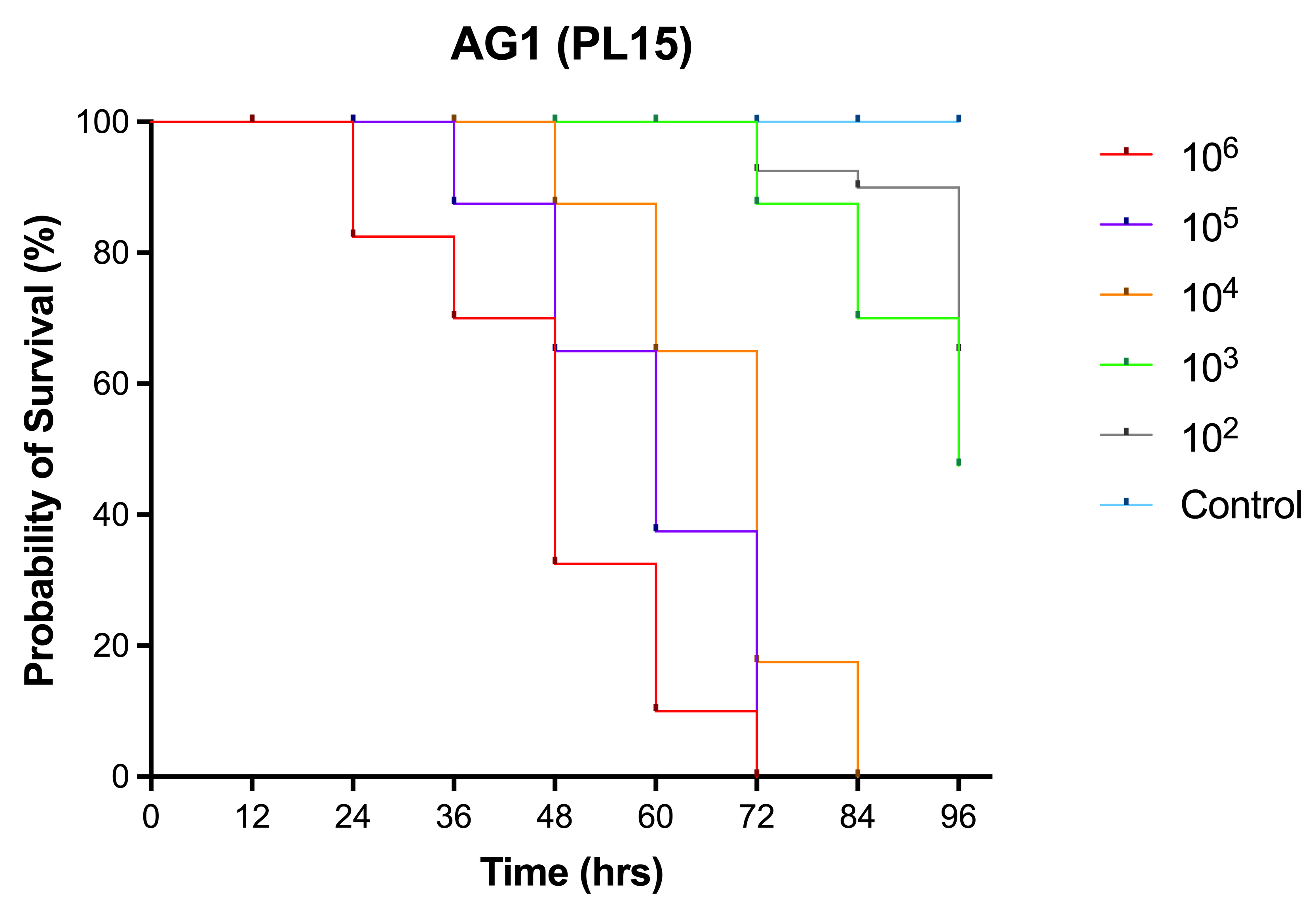

Supplement: S1 File — (ZIP) [file pone.0331862.s002.zip › S1 File/2B.tif]

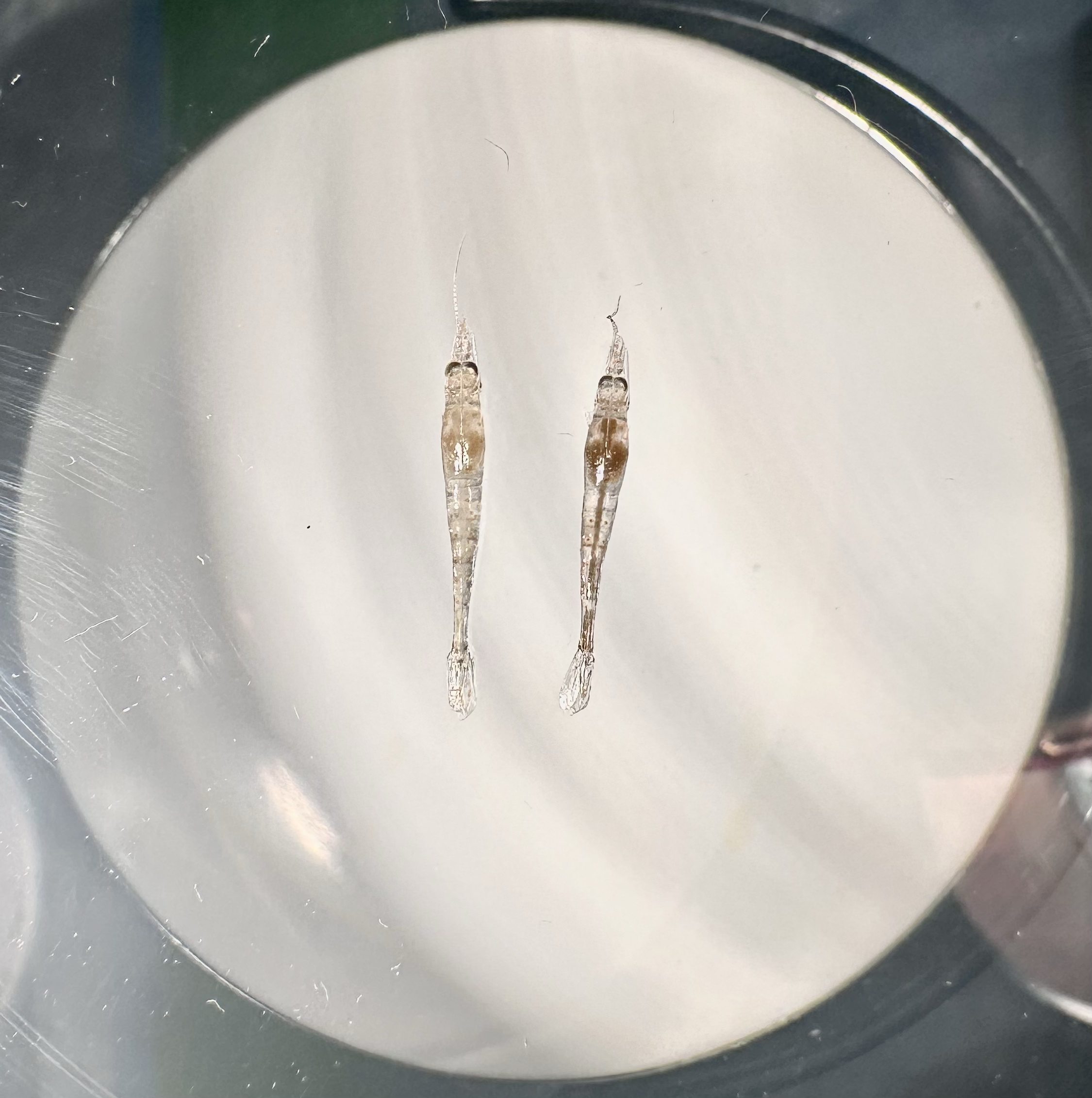

Supplement: S1 File — (ZIP) [file pone.0331862.s002.zip › S1 File/2C.tif]

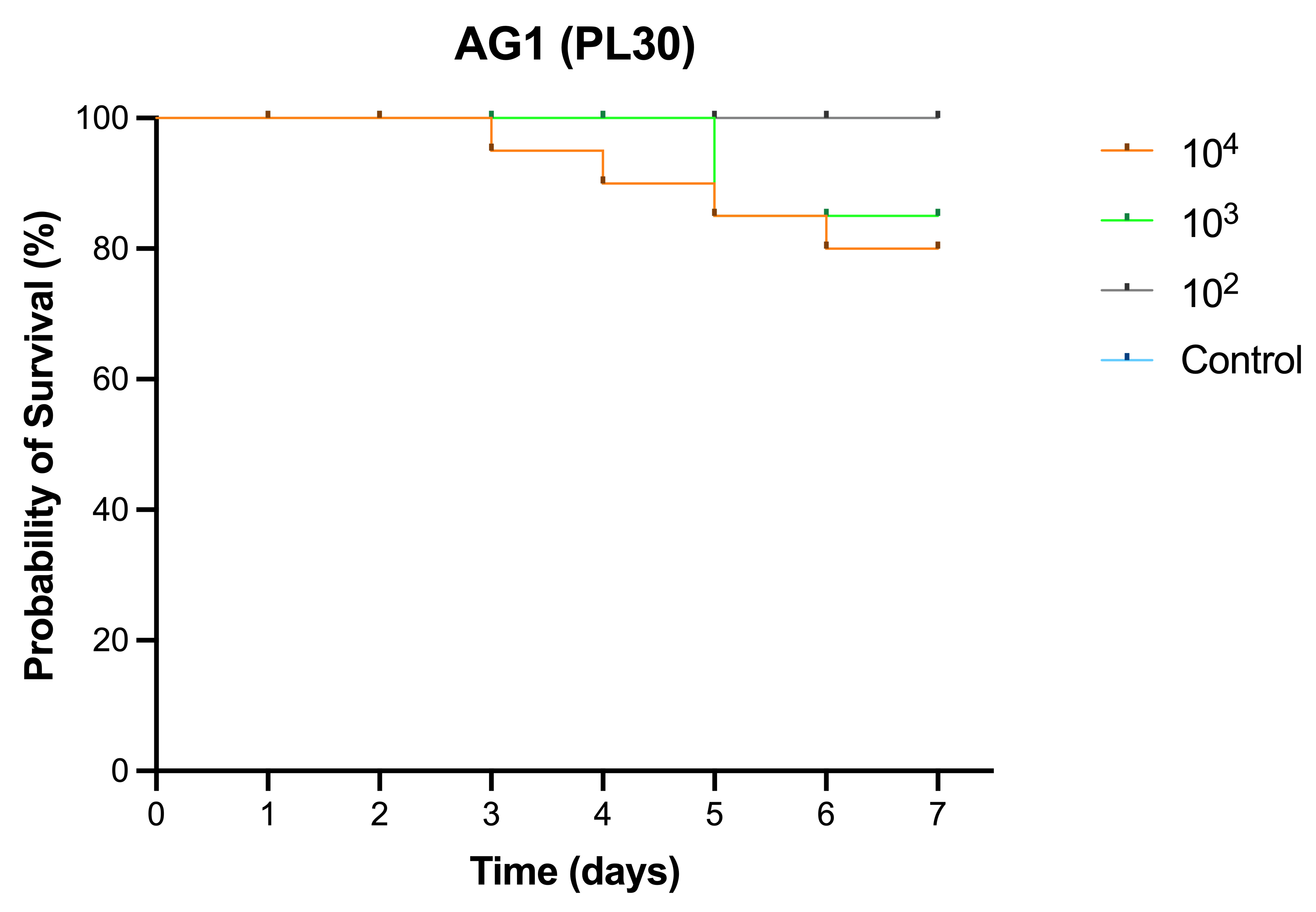

Supplement: S1 File — (ZIP) [file pone.0331862.s002.zip › S1 File/3.tif]

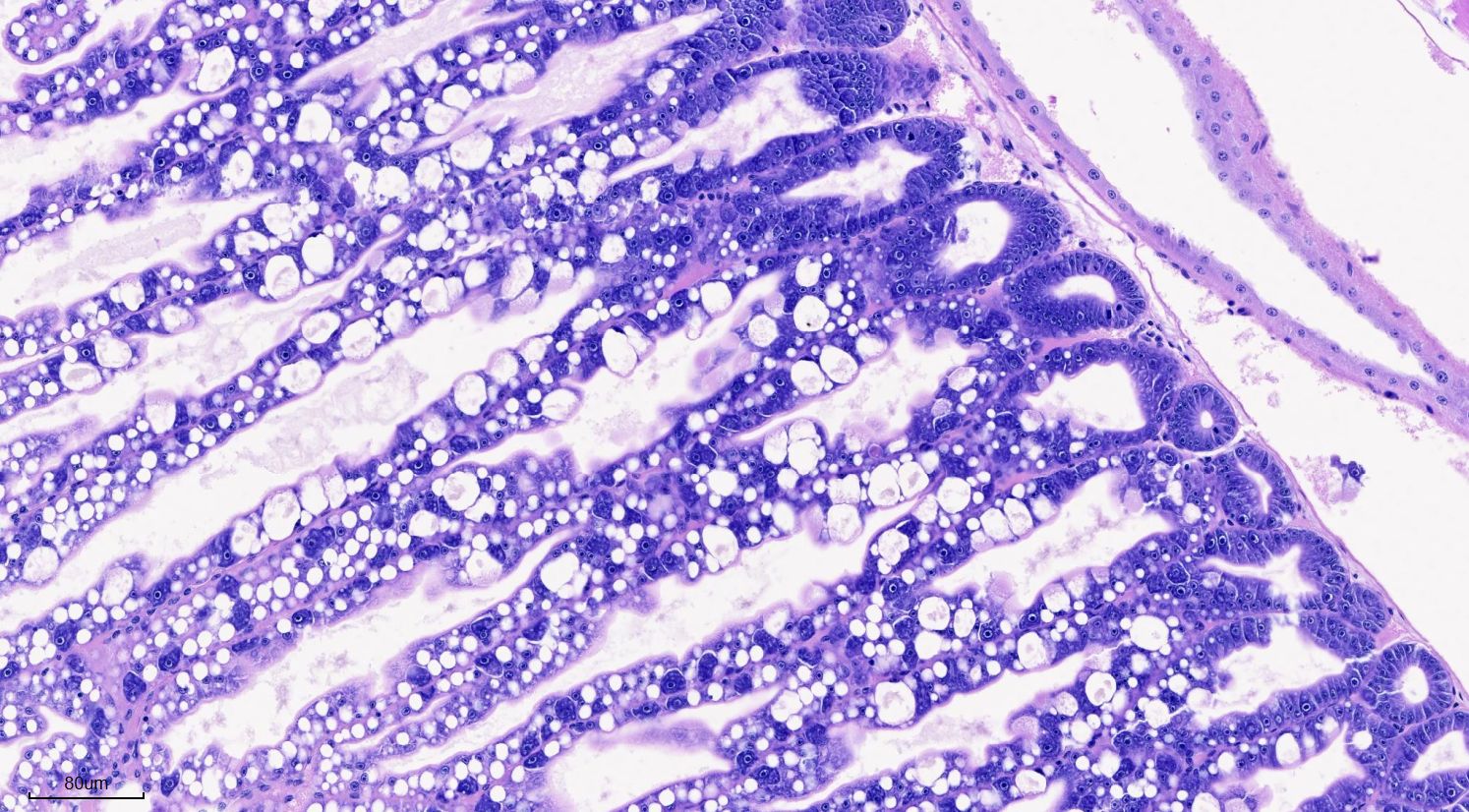

Supplement: S1 File — (ZIP) [file pone.0331862.s002.zip › S1 File/4A.tif]

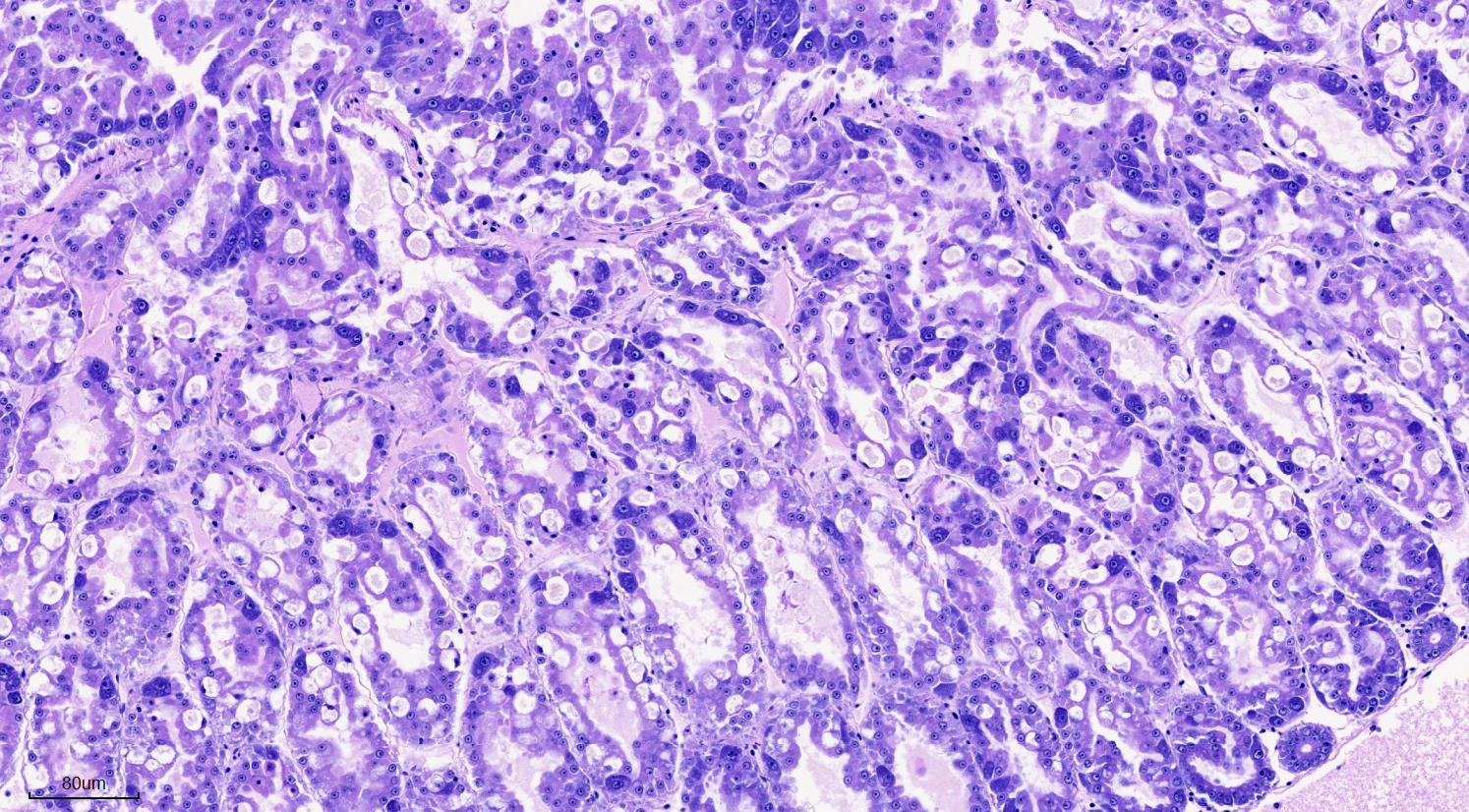

Supplement: S1 File — (ZIP) [file pone.0331862.s002.zip › S1 File/4B.tif]

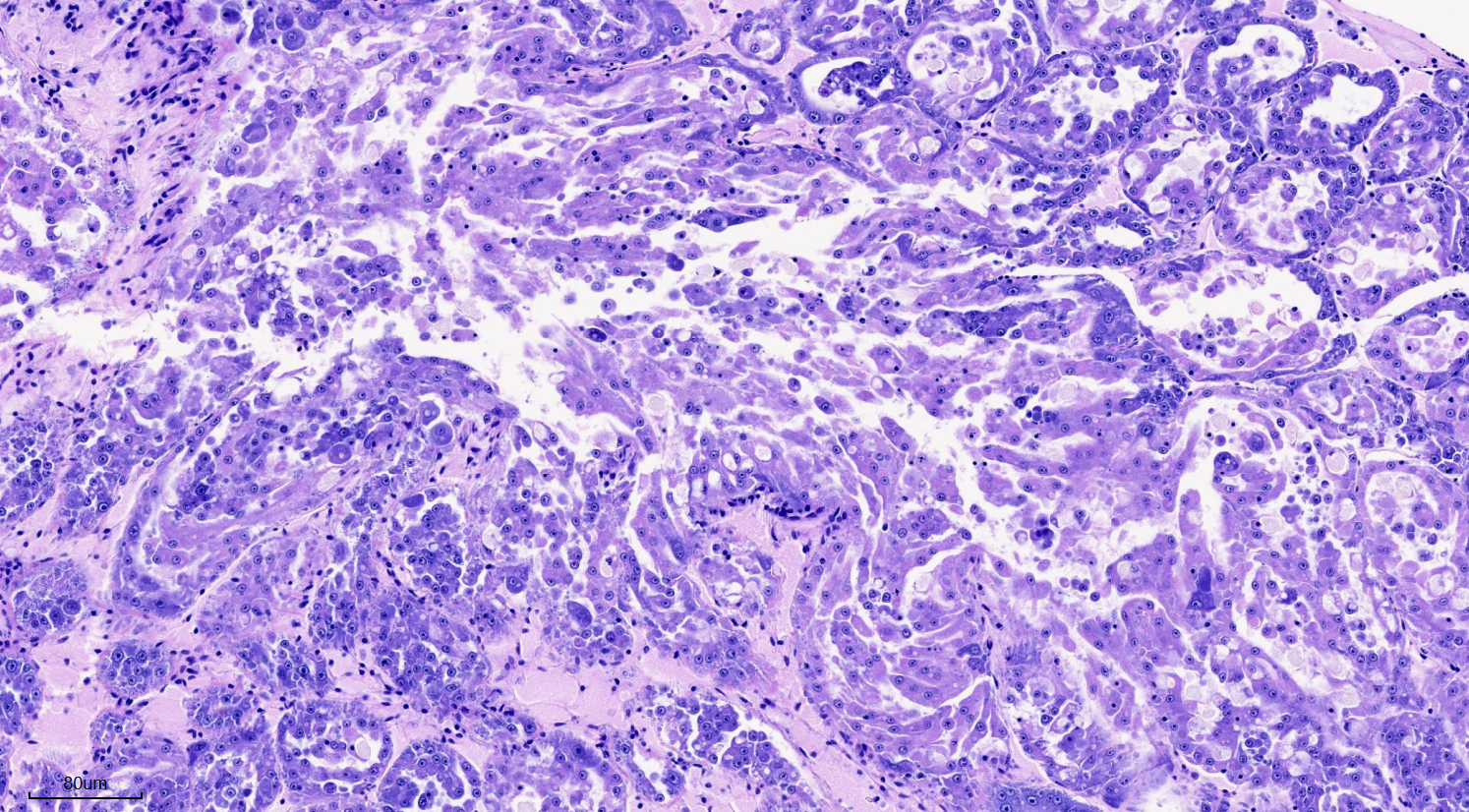

Supplement: S1 File — (ZIP) [file pone.0331862.s002.zip › S1 File/4C.tif]

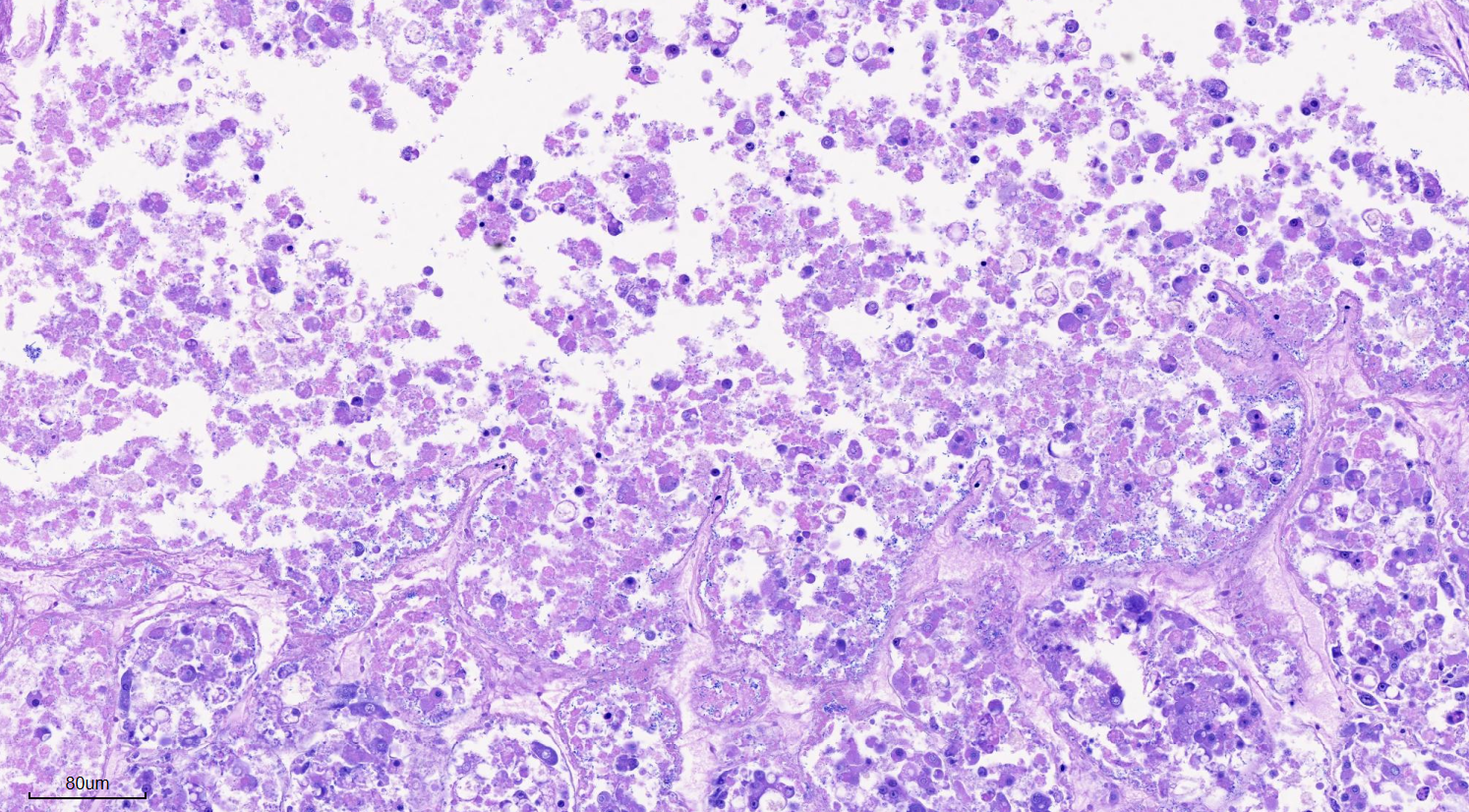

Supplement: S1 File — (ZIP) [file pone.0331862.s002.zip › S1 File/4D.tif]

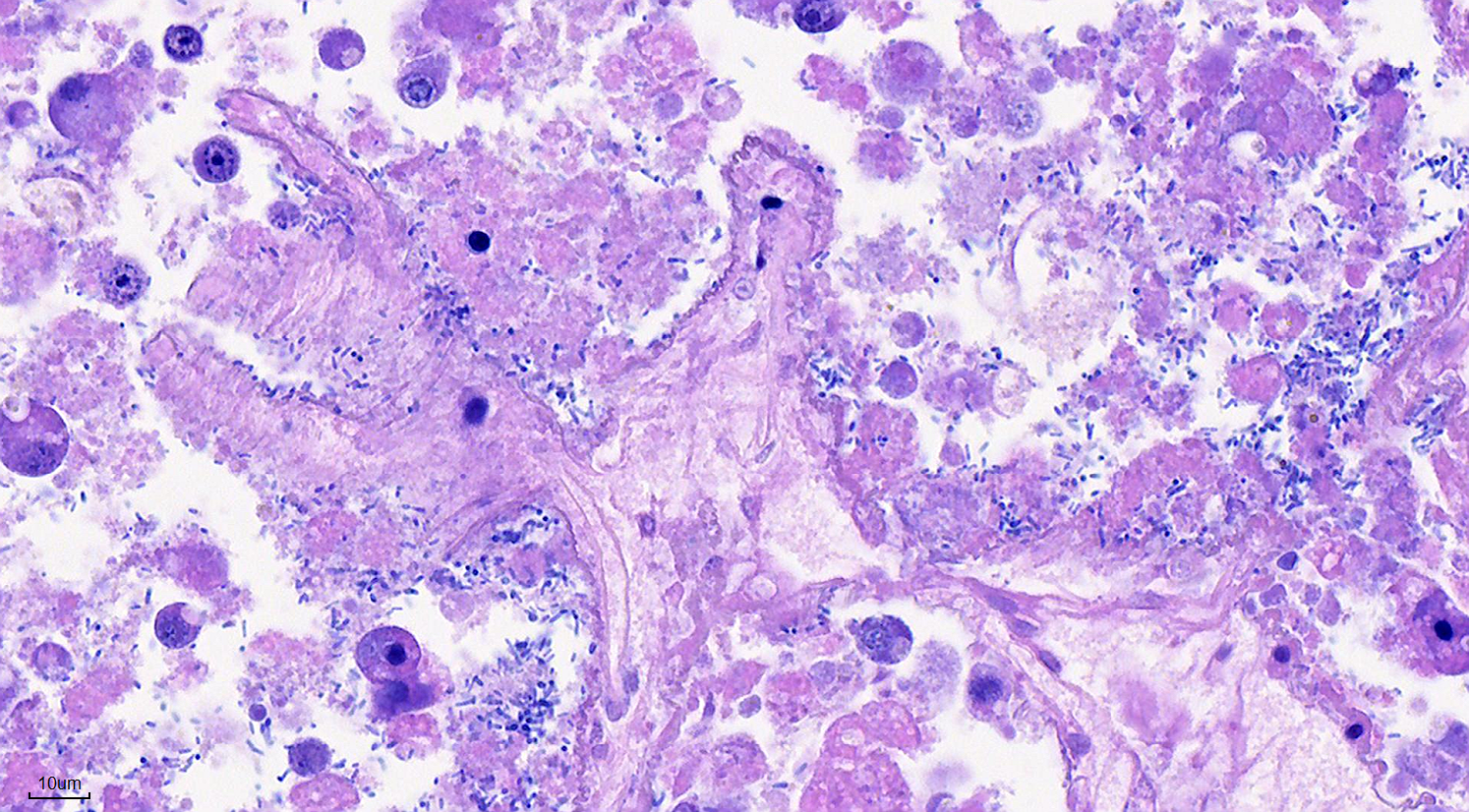

Supplement: S1 File — (ZIP) [file pone.0331862.s002.zip › S1 File/4E.tif]

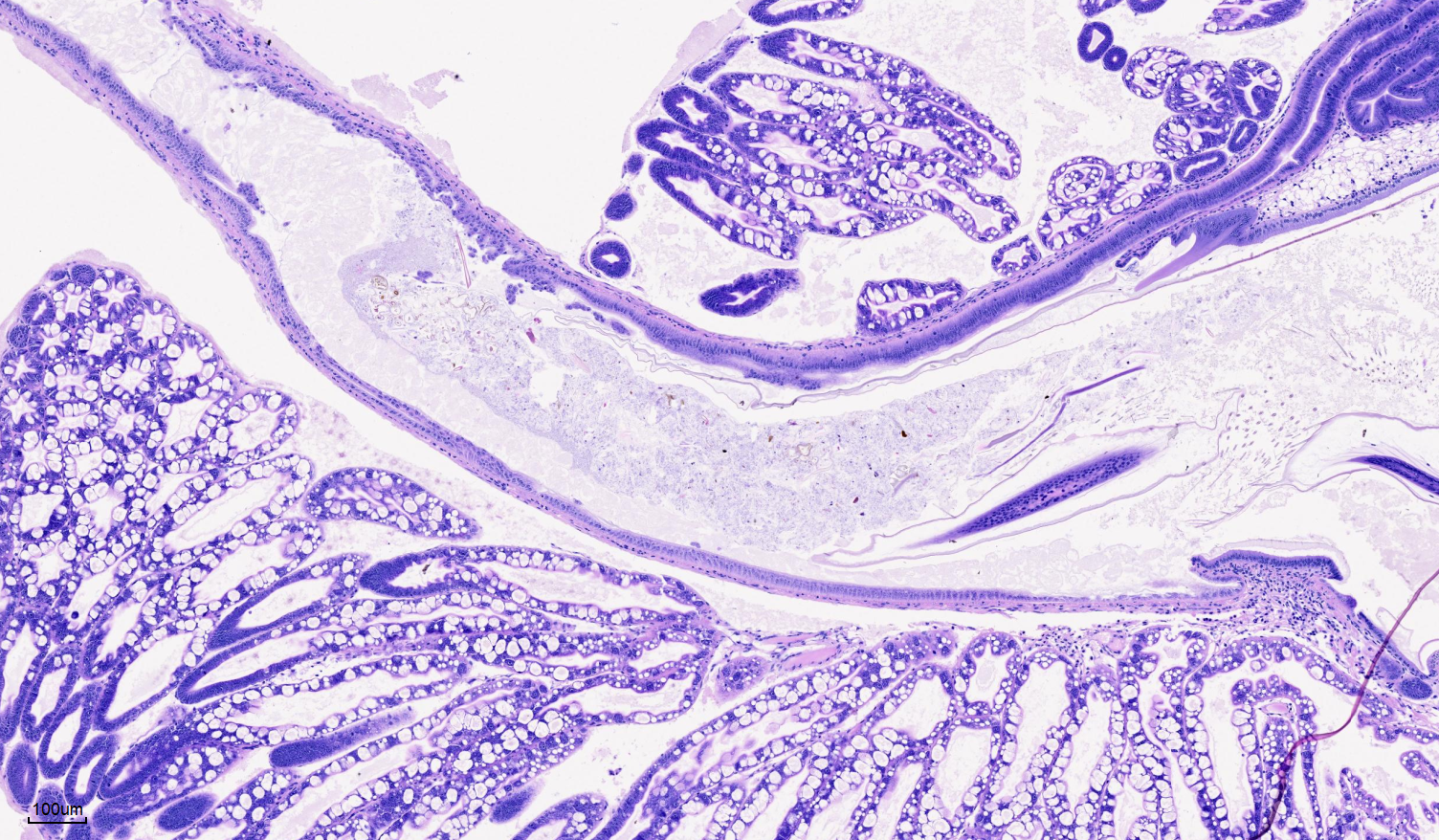

Supplement: S1 File — (ZIP) [file pone.0331862.s002.zip › S1 File/5A.tif]

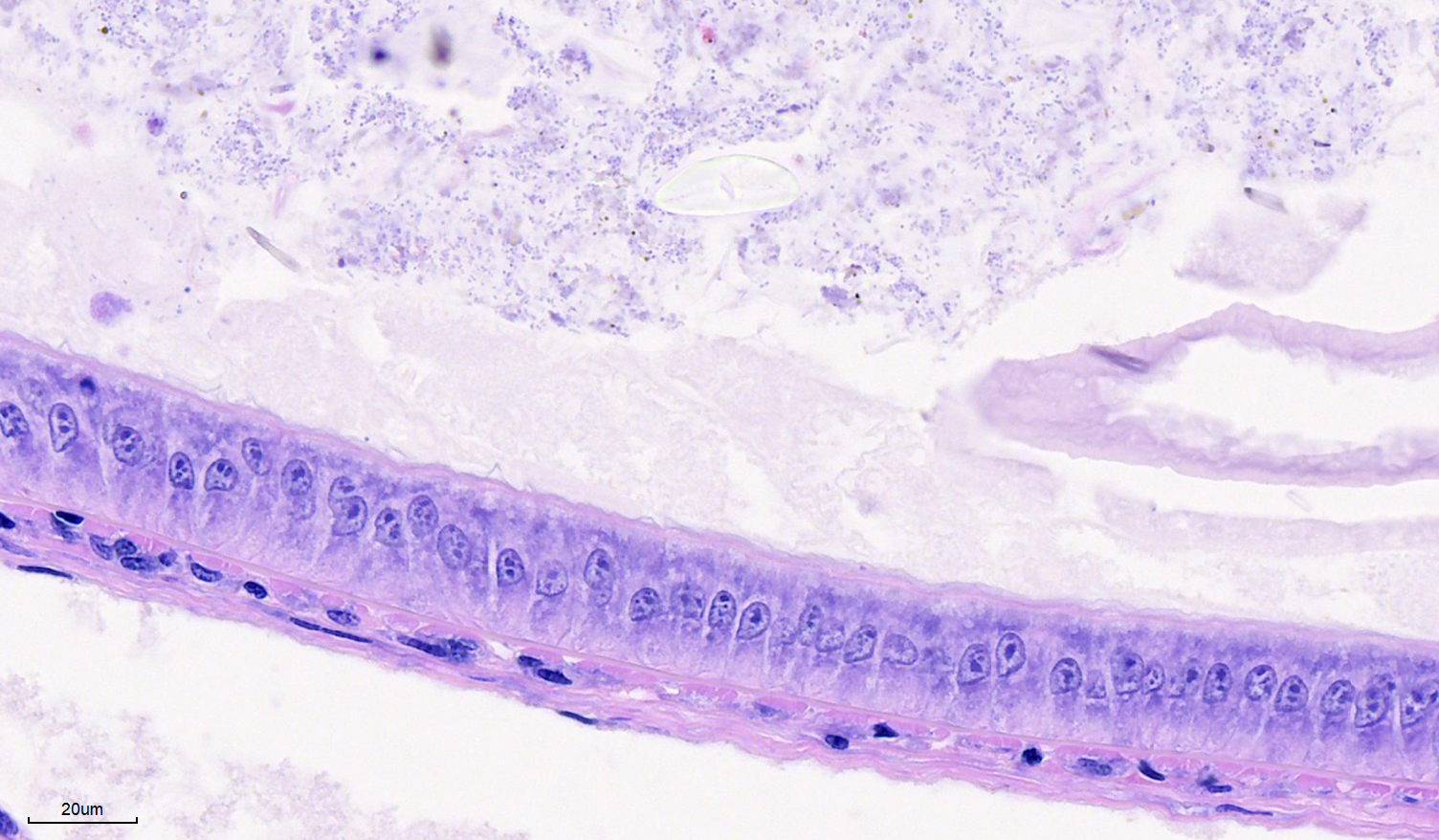

Supplement: S1 File — (ZIP) [file pone.0331862.s002.zip › S1 File/5B.tif]

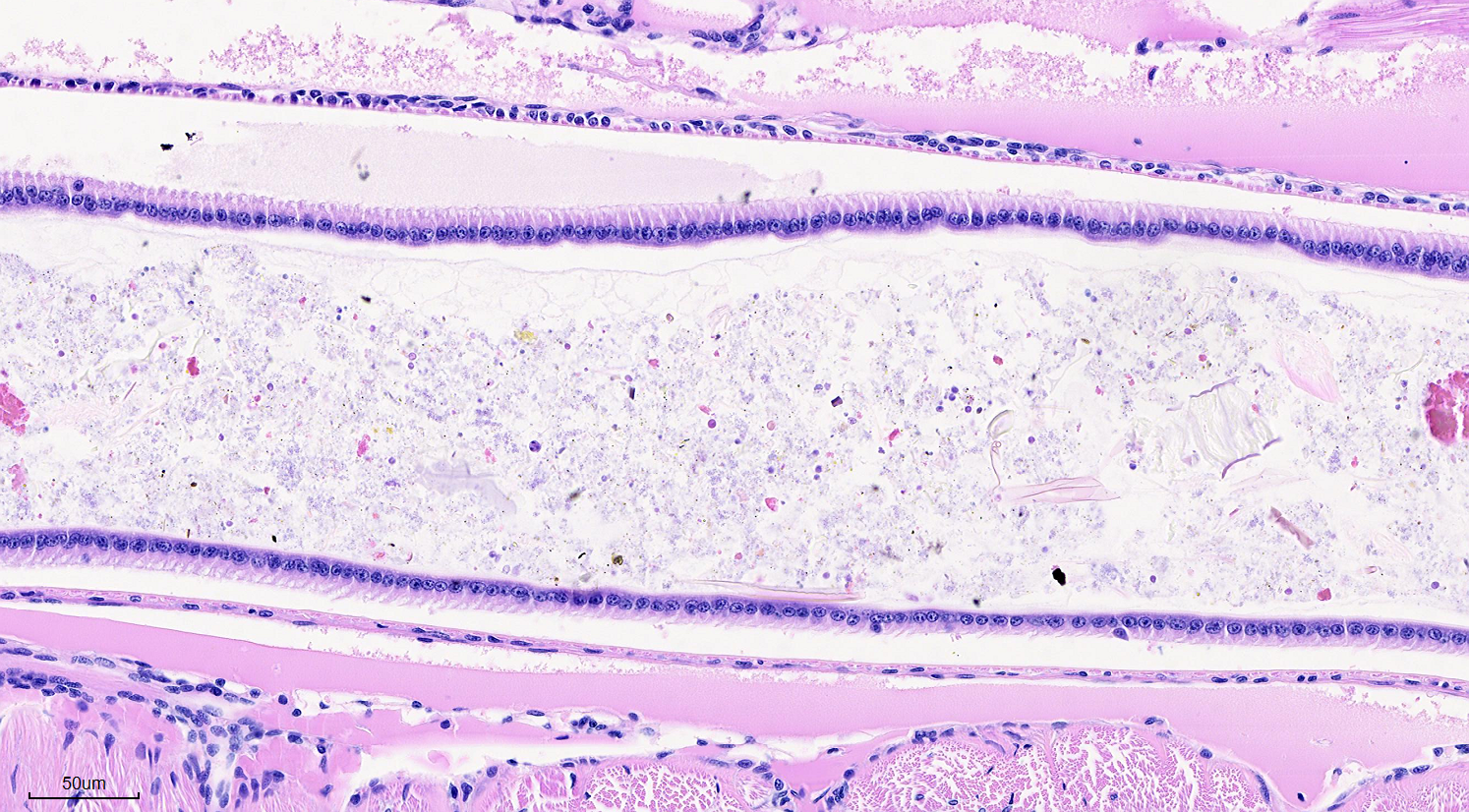

Supplement: S1 File — (ZIP) [file pone.0331862.s002.zip › S1 File/5C1.tif]

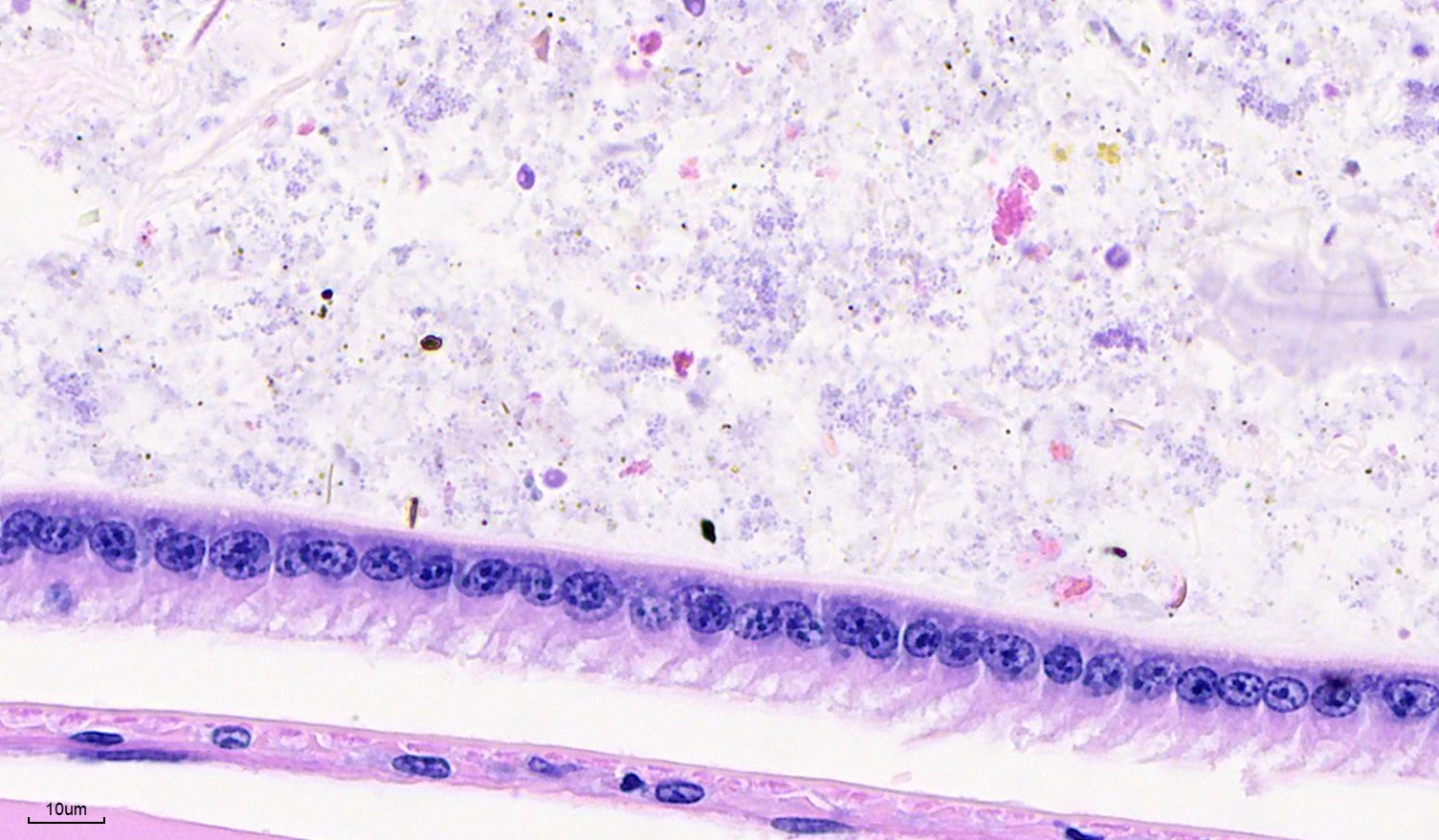

Supplement: S1 File — (ZIP) [file pone.0331862.s002.zip › S1 File/5C2.tif]

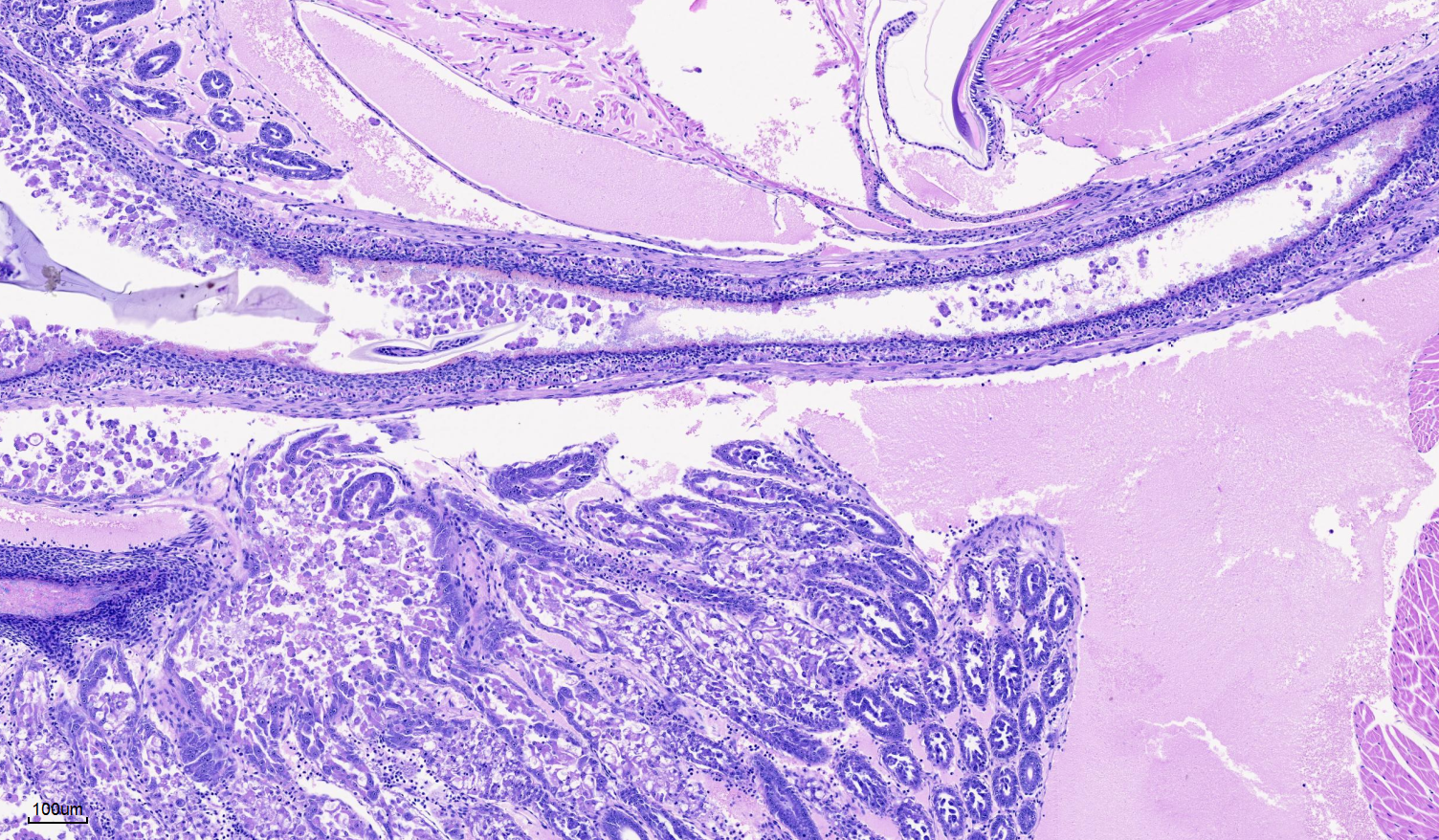

Supplement: S1 File — (ZIP) [file pone.0331862.s002.zip › S1 File/5D.tif]

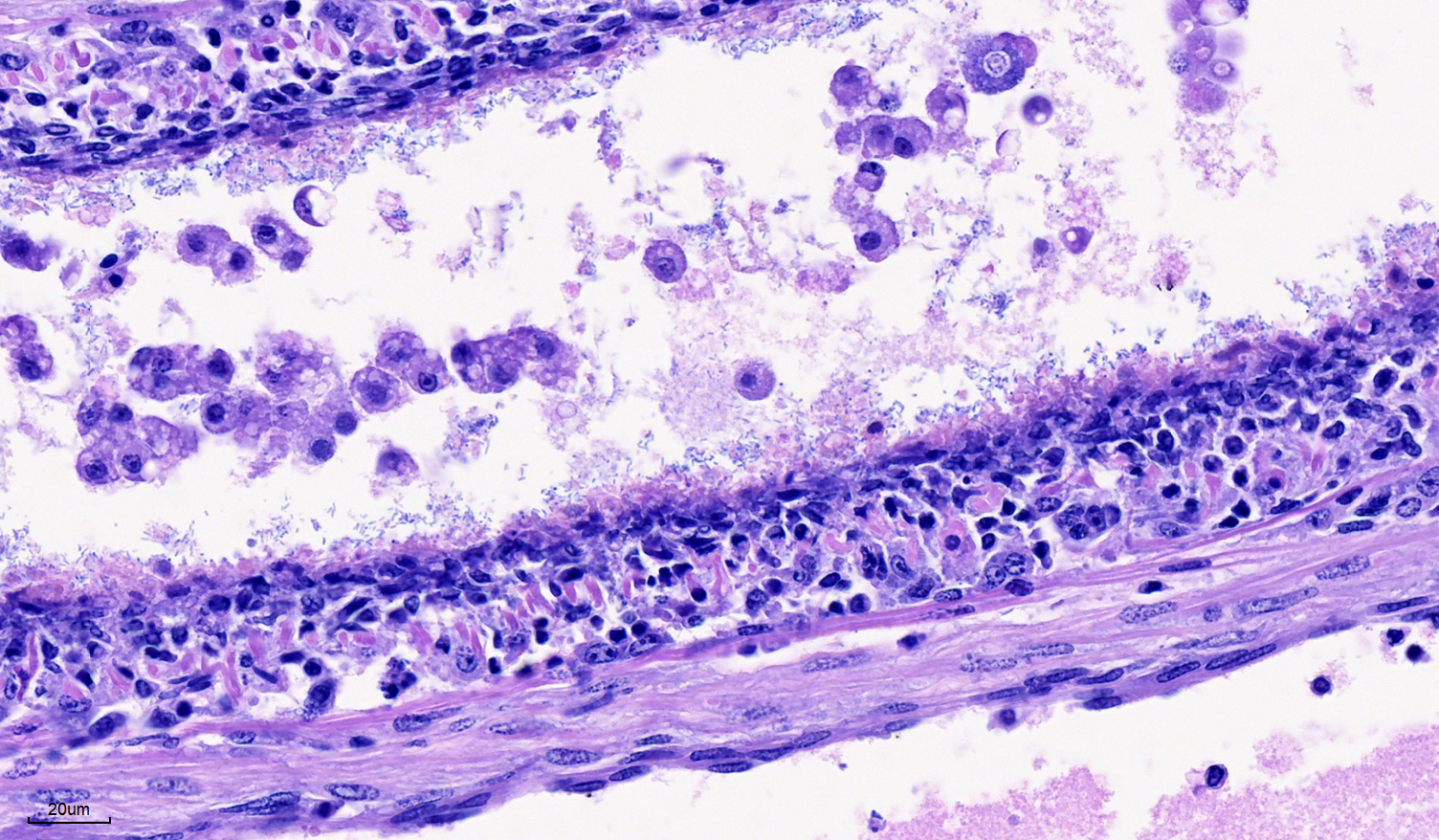

Supplement: S1 File — (ZIP) [file pone.0331862.s002.zip › S1 File/5E.tif]

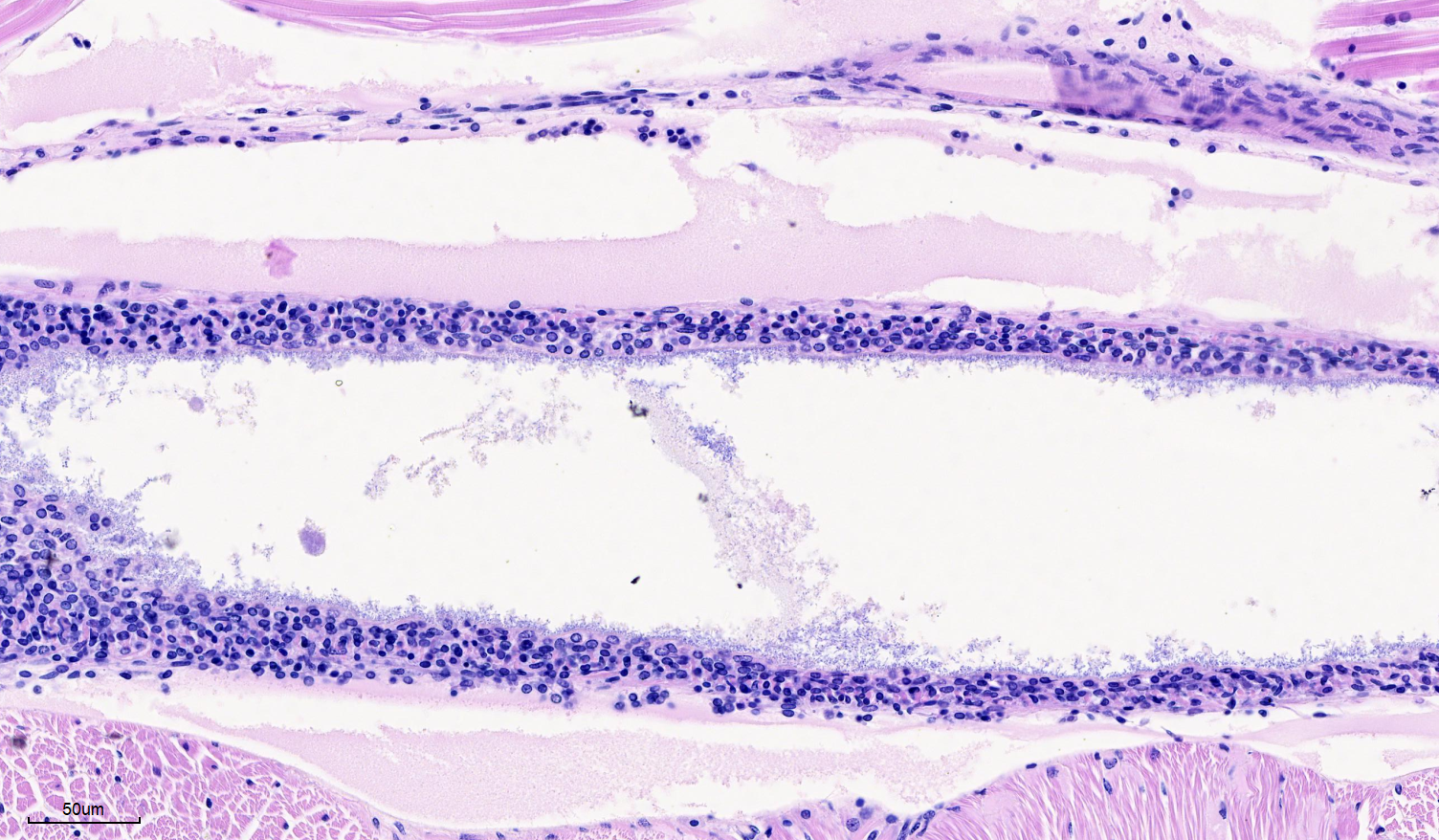

Supplement: S1 File — (ZIP) [file pone.0331862.s002.zip › S1 File/5F1.tif]

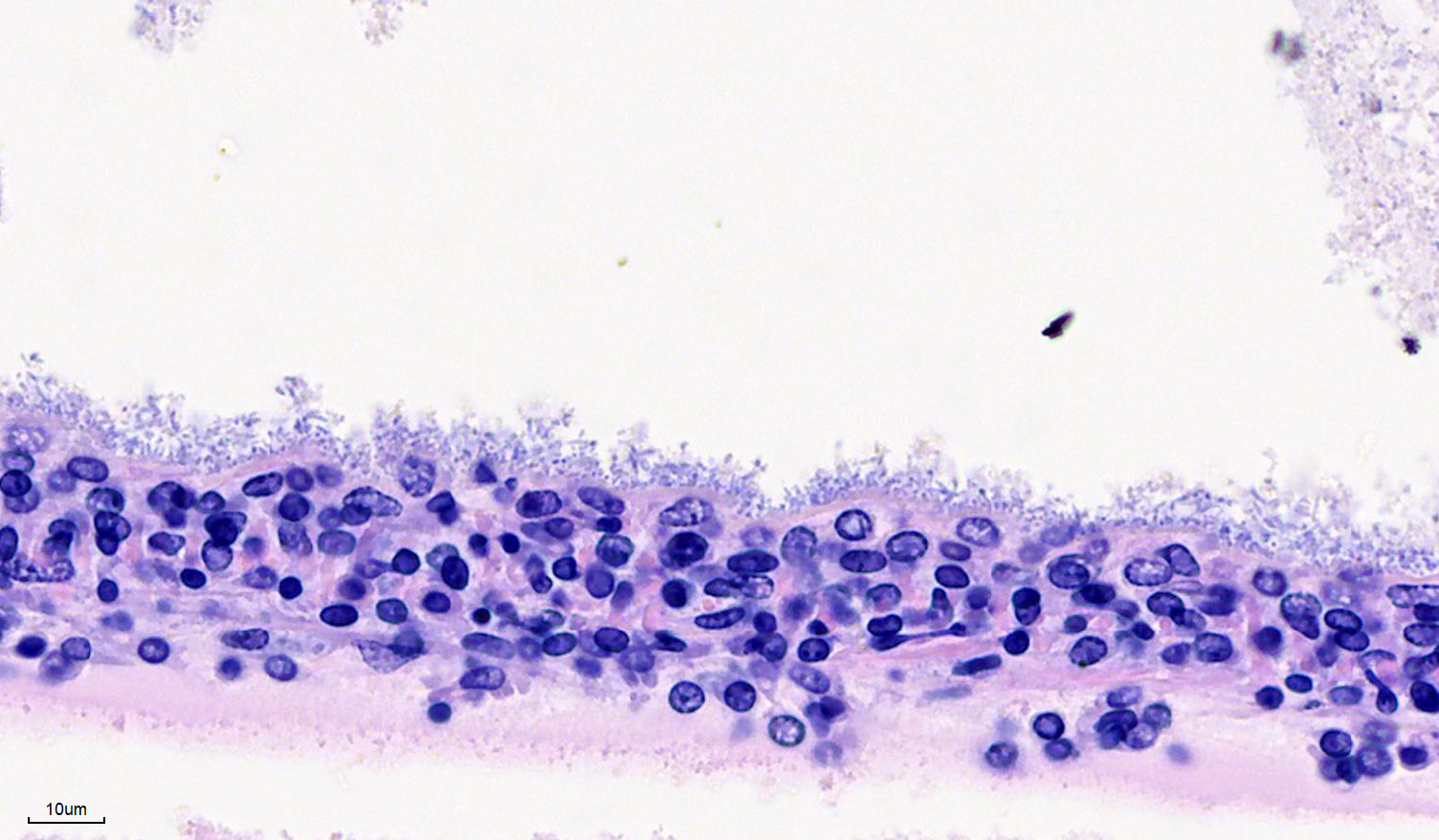

Supplement: S1 File — (ZIP) [file pone.0331862.s002.zip › S1 File/5F2.tif]

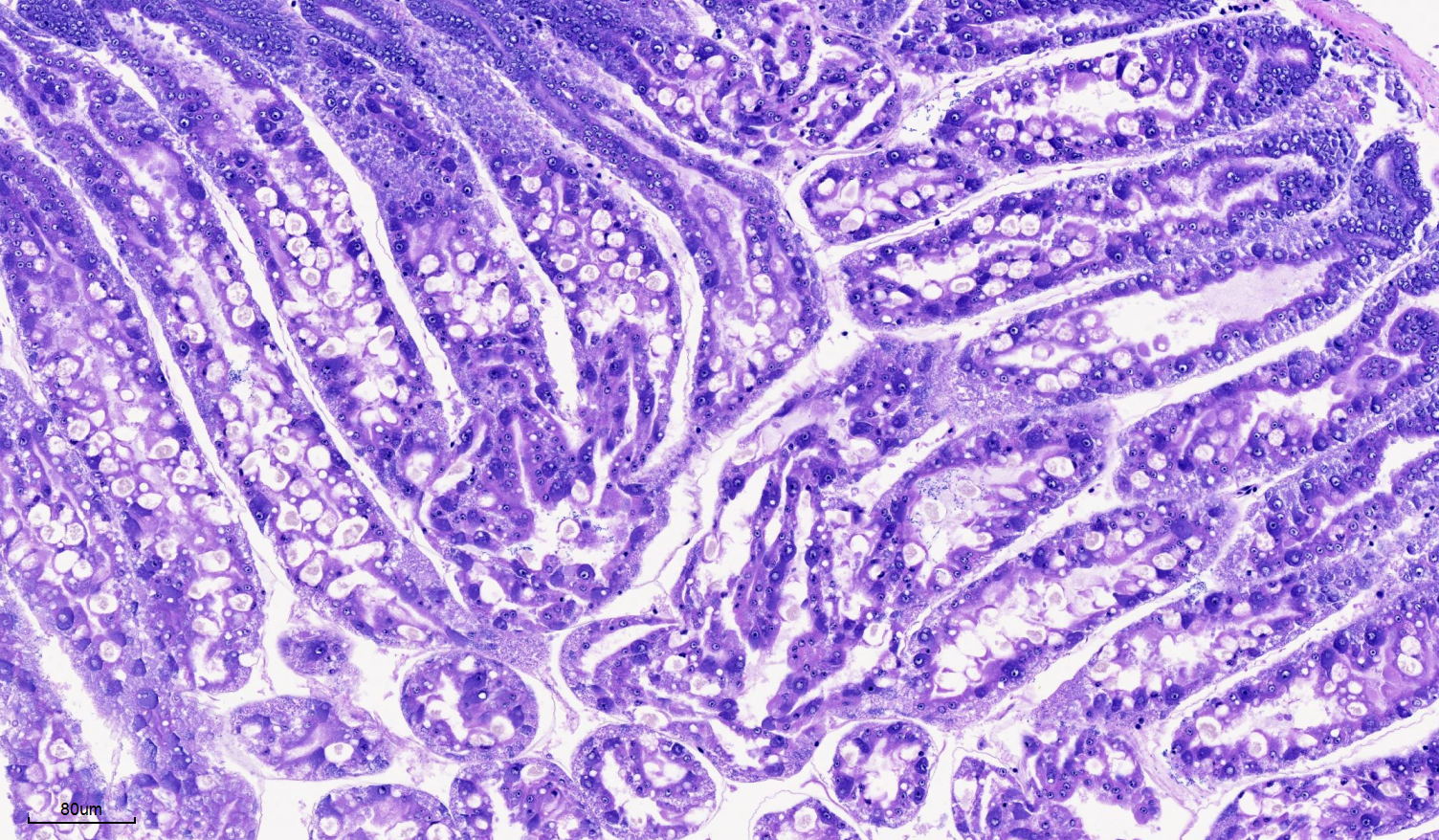

Supplement: S1 File — (ZIP) [file pone.0331862.s002.zip › S1 File/6A.tif]

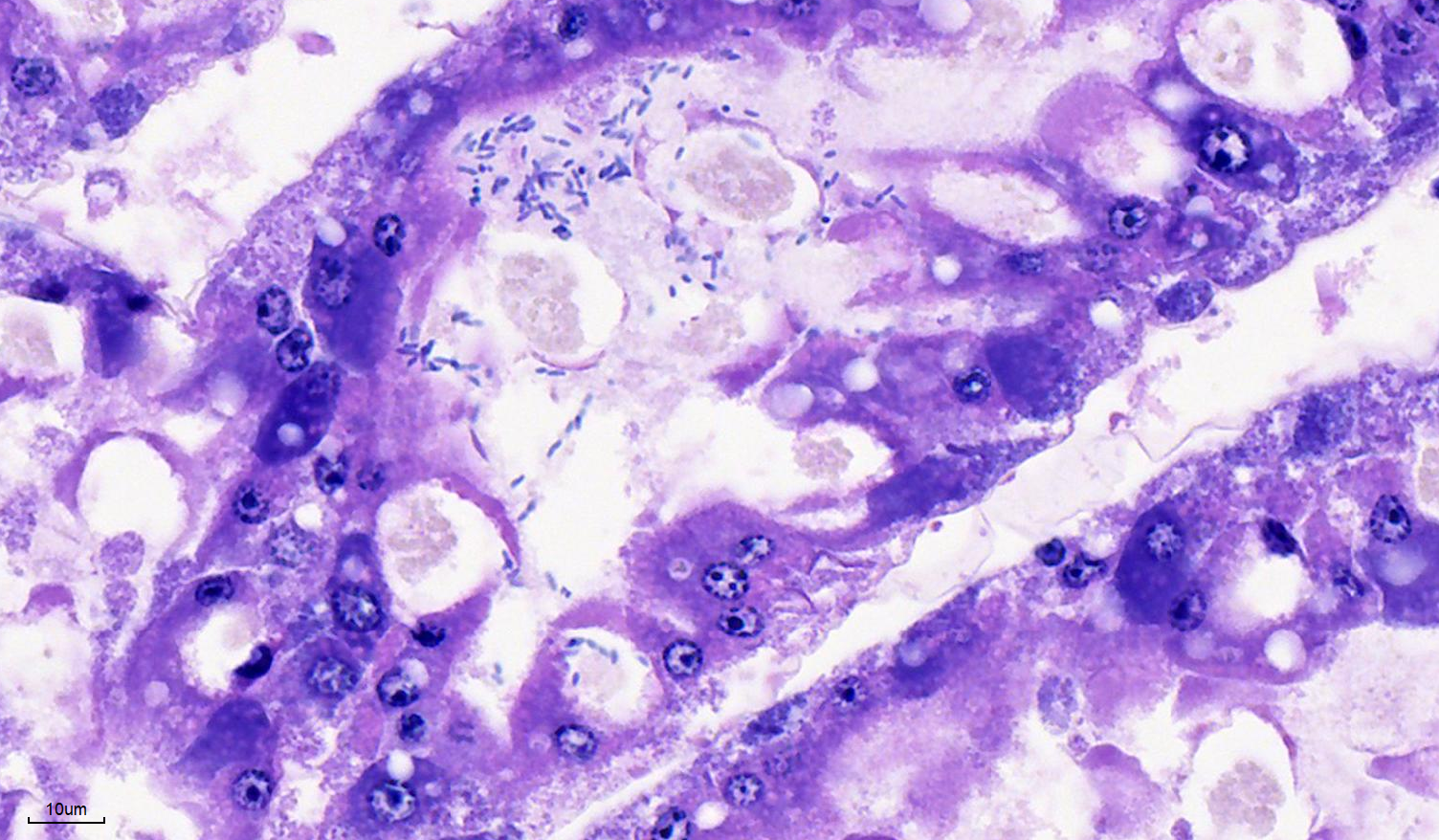

Supplement: S1 File — (ZIP) [file pone.0331862.s002.zip › S1 File/6B.tif]

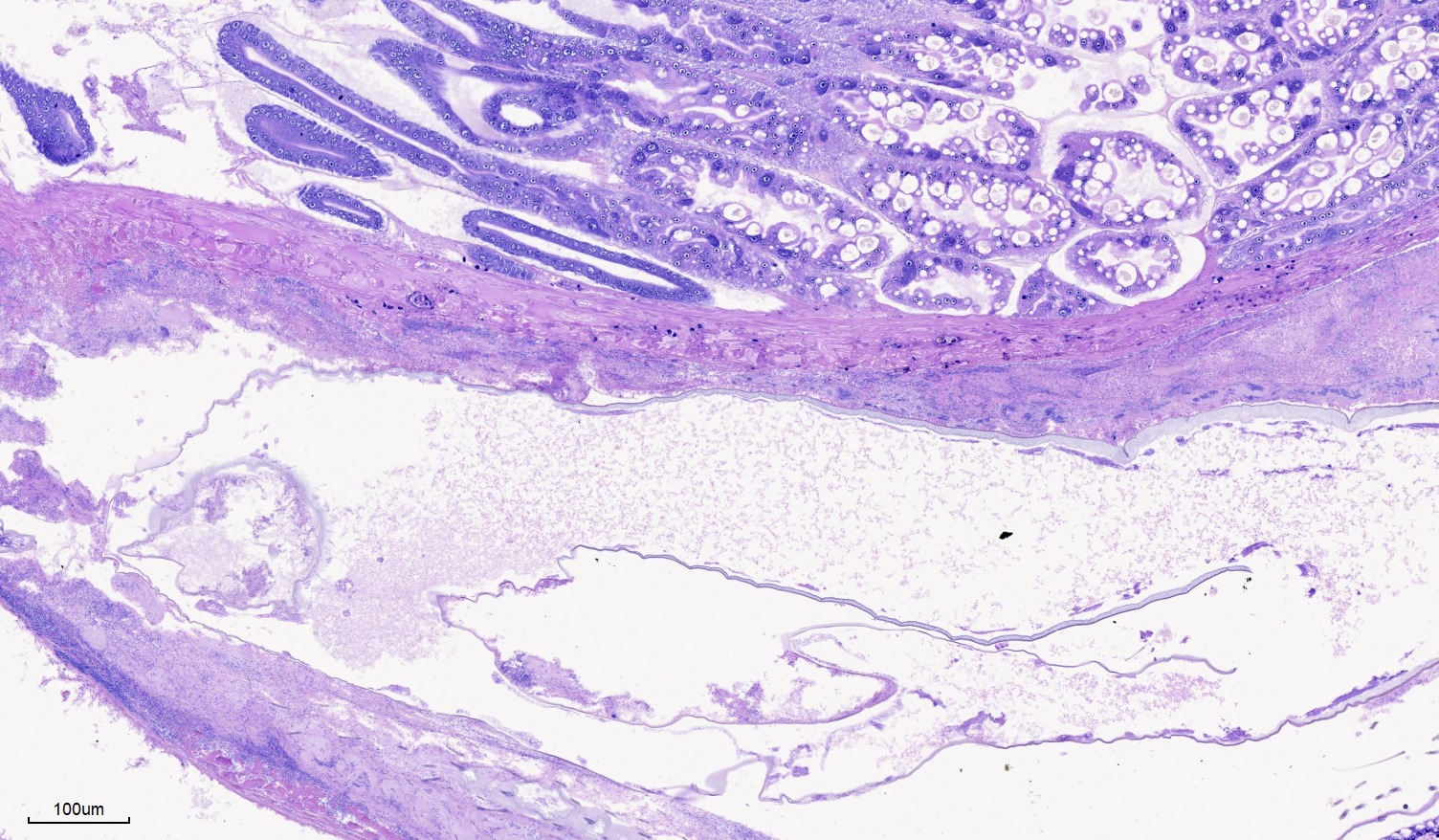

Supplement: S1 File — (ZIP) [file pone.0331862.s002.zip › S1 File/6C.tif]

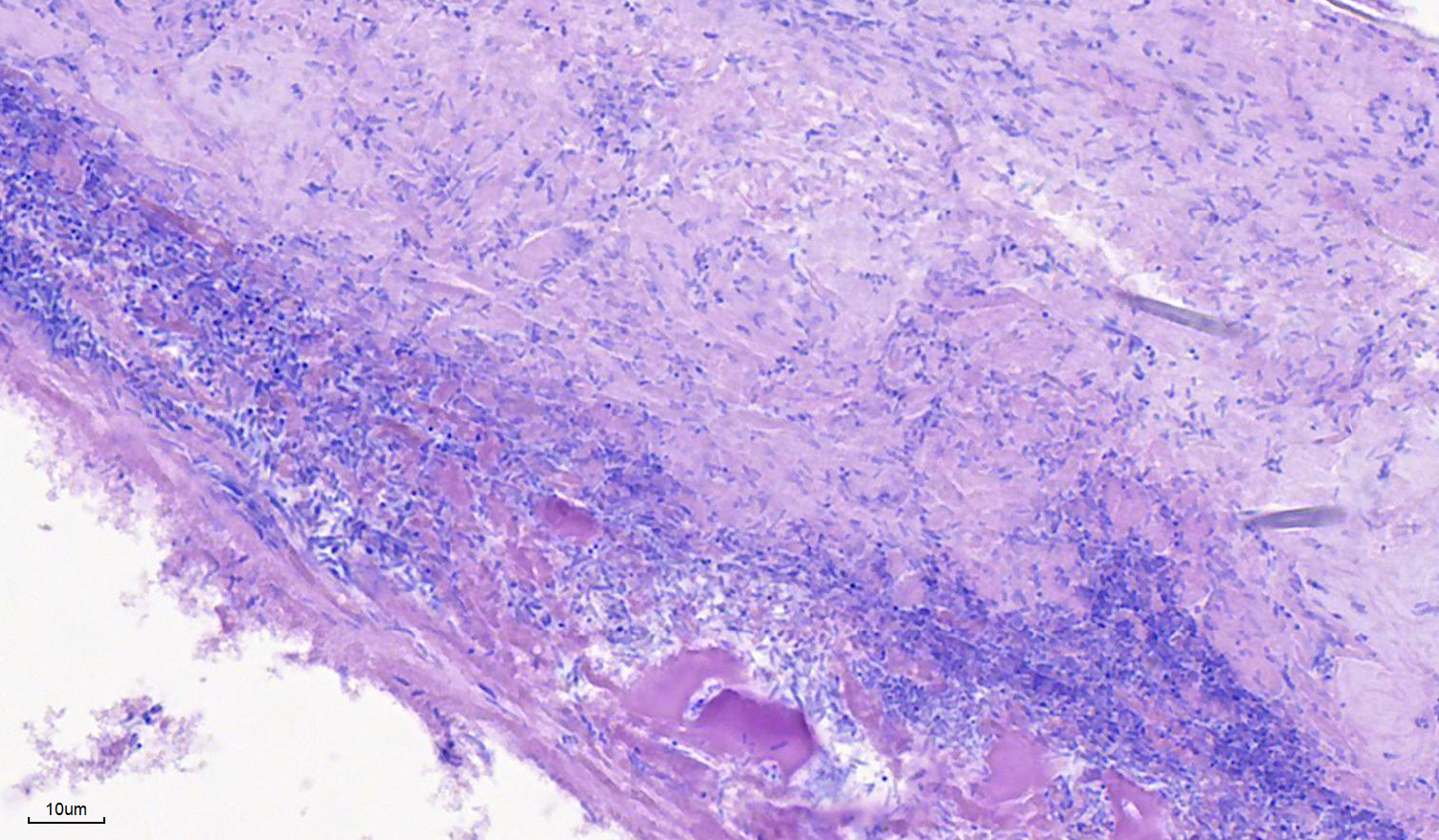

Supplement: S1 File — (ZIP) [file pone.0331862.s002.zip › S1 File/6D.tif]

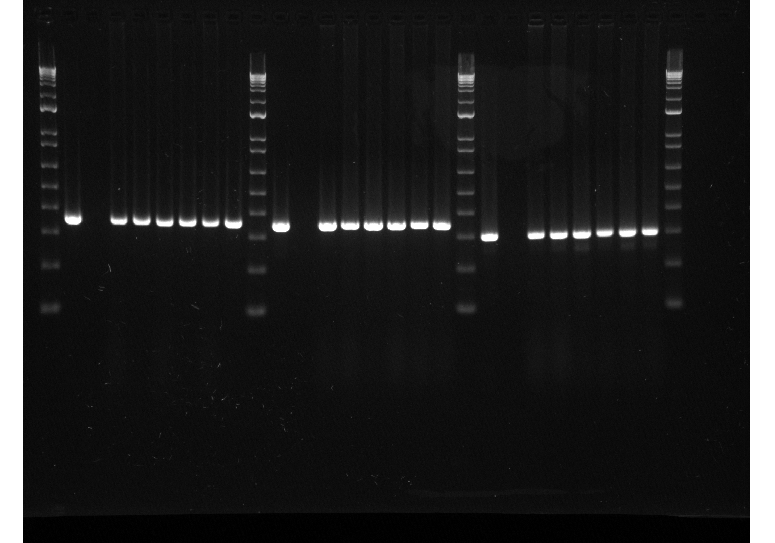

Supplement: S1 File — (ZIP) [file pone.0331862.s002.zip › S1 File/S1A.tif]

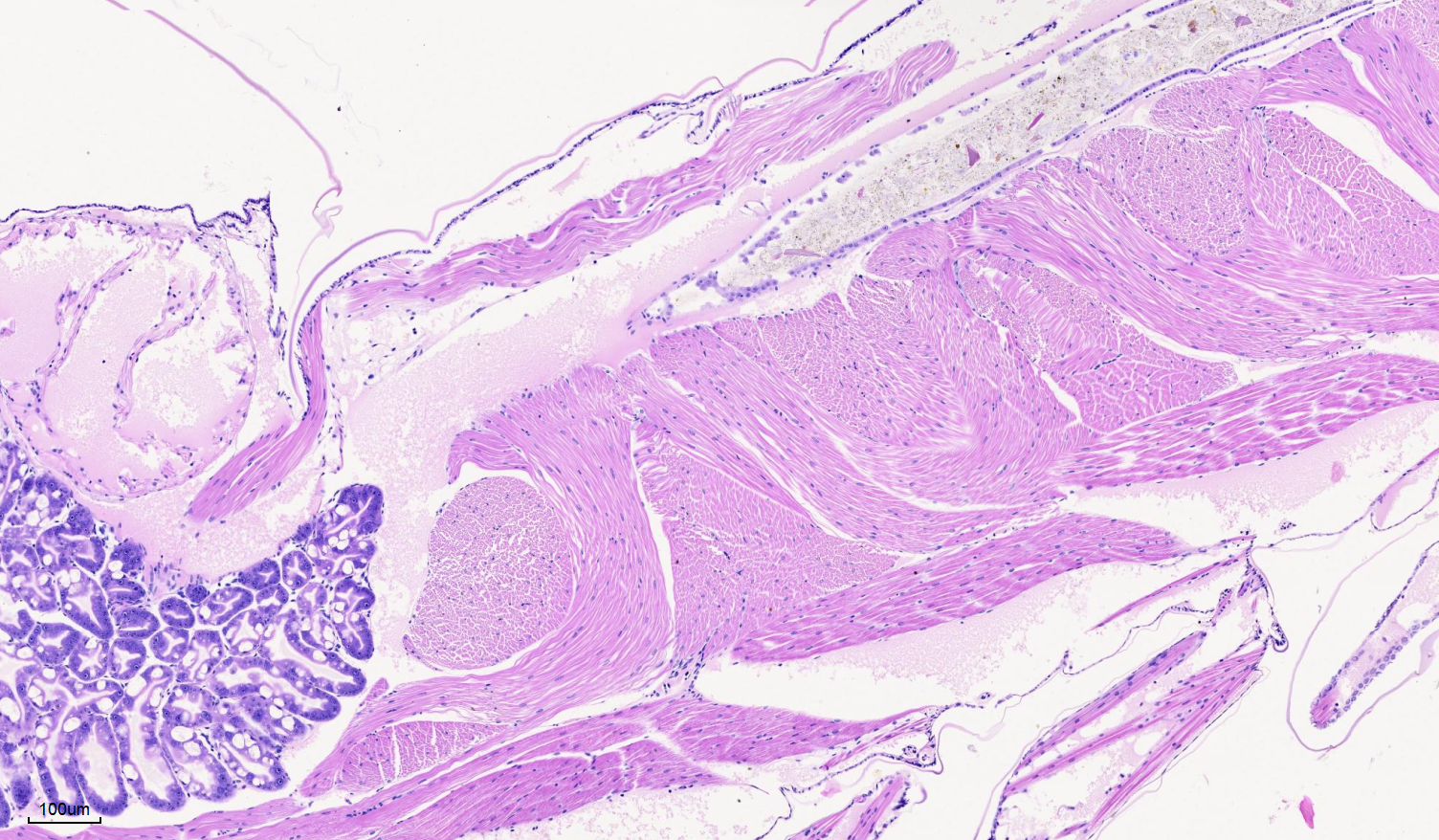

Supplement: S1 File — (ZIP) [file pone.0331862.s002.zip › S1 File/S1B.tif]

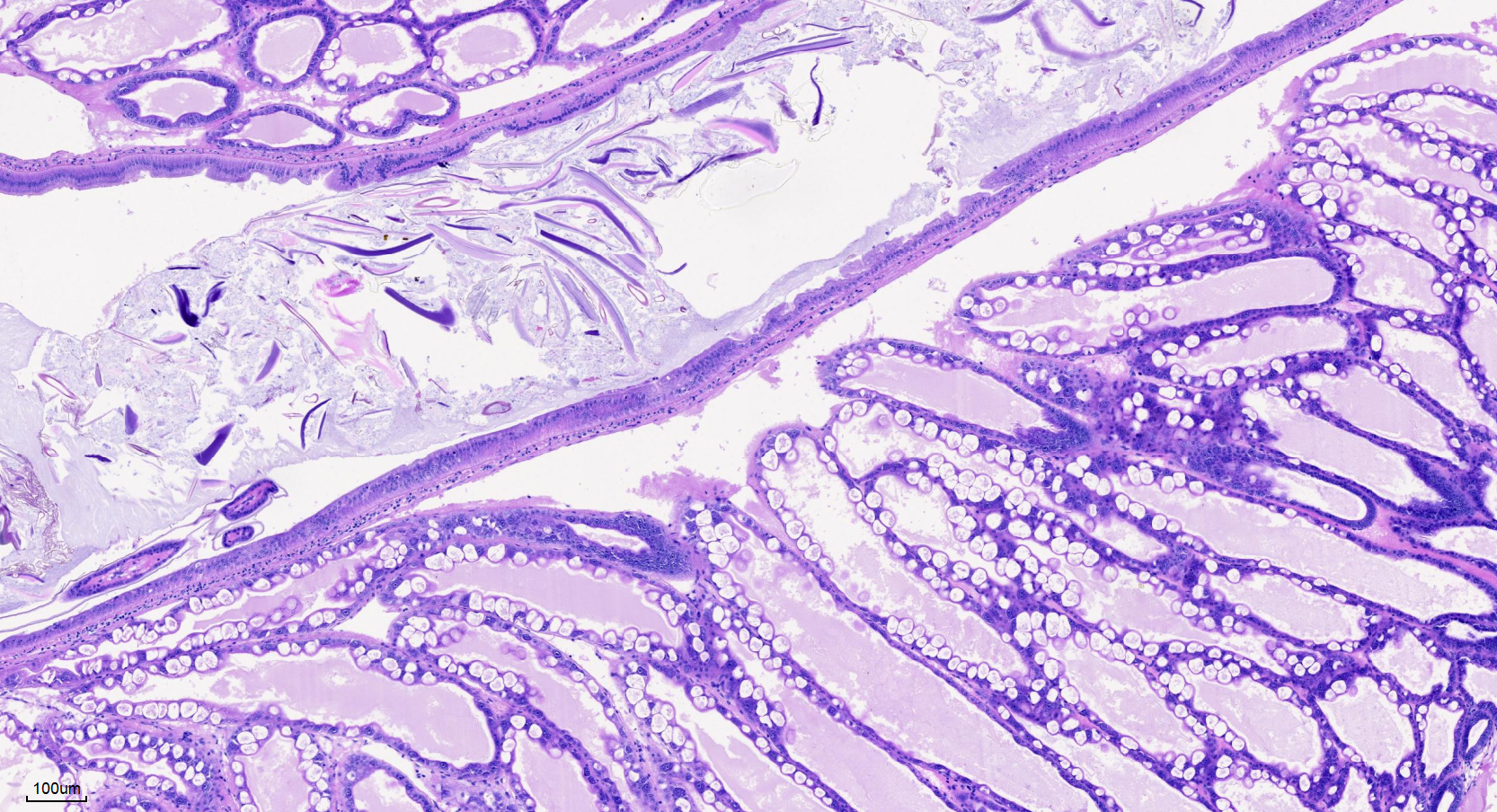

Supplement: S1 File — (ZIP) [file pone.0331862.s002.zip › S1 File/S1C.tif]
